# Adverse pregnancy and birth outcomes in women with biopsy-proven MASLD: a nationwide cohort study

**DOI:** 10.1016/j.eclinm.2025.103238

**Published:** 2025-05-09

**Authors:** Carole A. Marxer, Fahim Ebrahimi, David Bergman, Jiangwei Sun, Hannes Hagström, Marcus Thuresson, Olof Stephansson, Jonas F. Ludvigsson

**Affiliations:** a Department of Medical Epidemiology and Biostatistics, Karolinska Institutet, Stockholm, Sweden; b Department of Gastroenterology and Hepatology, University Digestive Health Care Center Basel – Clarunis, Basel, Switzerland; c Division of Hepatology, Department of Upper GI, Karolinska University Hospital, Stockholm, Sweden; d Department of Medicine, Huddinge, Karolinska Institutet, Stockholm, Sweden; e Statisticon AB, Uppsala, Sweden; f Department of Medicine, Solna, Karolinska Institutet, Stockholm, Sweden; g Department of Women's Health, Division of Obstetrics, Karolinska University Hospital, Stockholm, Sweden; h Department of Paediatrics, Örebro University Hospital, Örebro, Sweden; i Department of Medicine, Columbia University College of Physicians and Surgeons, New York, NY, USA

**Keywords:** MASLD, Pregnancy outcomes, Preterm birth, Cesarean section, Epidemiology

## Abstract

**Background:**

Metabolic dysfunction-associated steatotic liver disease (MASLD) has been linked to an increased risk of adverse pregnancy outcomes. We aimed to assess the roles of obesity, familial factors, and disease severity.

**Methods:**

Nationwide cohort study in Sweden (1992–2017) including 240 births (162 women) with biopsy-proven MASLD vs. 1140 matched reference births (1138 women). Multivariable conditional logistic regression determined adjusted odds ratios (aORs) for adverse pregnancy outcomes.

**Findings:**

Preterm birth occurred in 40 (16.7%) births of women with MASLD compared to 53 (4.7%) in reference births, yielding an aOR of 3.41 (95% CI = 1.98–5.88), which remained increased when compared with overweight/obese women without known MASLD (aOR = 4.60, 95% CI = 2.00–10.60). The association was observed for both medically indicated preterm birth (aOR = 11.90, 95% CI = 2.46–57.59) and spontaneous preterm birth (aOR = 2.42, 95% CI = 1.16–5.04). The aOR of preterm birth did not increase with MASLD severity (MASH without fibrosis/noncirrhotic fibrosis/cirrhosis: aOR = 1.53, 95% CI = 0.23–10.02), but 95% CIs were wide. MASLD was linked to increased odds of cesarean section (aOR = 1.63, 95% CI = 1.17–2.27), but not when compared with overweight/obese reference women (aOR = 1.20, 95% CI = 0.77–1.86). Our results indicate comparable Apgar scores, and similar rates of congenital malformation, stillbirth and neonatal death in both groups. Main results were confirmed in sibling analyses (i.e., when comparing to births of full female siblings of the mothers).

**Interpretation:**

MASLD is a risk factor for preterm birth, independent of obesity and familial factors. MASLD however does not indicate more stillbirths and neonatal deaths. We did not find more adverse pregnancy outcomes with increasing MASLD severity.

**Funding:**

10.13039/100000001Swiss National Science Foundation (P500PM_217670, P500PM_210866), European Crohn's and Colitis Organisation and, The 10.13039/501100003748Swedish Society for Medical Research (PG-23-0315-H-02) and 10.13039/501100004047Karolinska Institute.

**Research in context**

**Evidence before this study**

Metabolic dysfunction-associated steatotic liver disease (MASLD) is associated with an increased risk of several adverse pregnancy outcomes, but the roles of obesity, familial factors, and disease severity are under investigated.

**Added value of this study**

We found a more than tripled risk of preterm birth in newborns to mothers with MASLD, which was independent of obesity and familial confounders (i.e., early environmental factors and shared genetics), and we did not find more preterm births with increasing disease severity. Previous studies lacked comprehensive data on maternal BMI, sibling health records, and disease severity, making this study the first to provide such evidence.

**Implications of all the available evidence**

This key finding may influence the European and American clinical practice guidelines for managing MASLD in pregnancy, which currently recommend monitoring risks related to obesity and diabetes (risk factors of MASLD). The latest suggestion may be revised to recommend monitoring risks associated with MASLD itself and to prompt changes in preconception counseling. Lastly, our study highlights the importance of further studies evaluating the underlying mechanisms driving this observed association and underscores the need for clinical vigilance among offspring born to mothers with MASLD.

## Introduction

Metabolic dysfunction-associated steatotic liver disease (MASLD, formerly known as non-alcoholic fatty liver disease, NAFLD^1^) is the most common chronic liver disease in the world, with an estimated global prevalence of 32%^2^ (23% in Sweden^3^). The prevalence is estimated to be around 10% in women of childbearing age (20–40 years),^4^ and varies depending on factors such as the rate of obesity and type 2 diabetes in the general population,^5^ as well as age and mode of diagnosis (non-invasive tests, ultrasound and/or biopsy).^6^

Previous observational studies have collectively shown that MASLD in pregnant women is associated with a substantial increase in maternal metabolic complications such as gestational diabetes, gestational hypertension, and pre-eclampsia, however these findings may be in part a consequence of obesity, rather than MASLD.^7^, ^8^, ^9^, ^10^, ^11^, ^12^, ^13^ Evidence on other pregnancy and birth outcomes (e.g., preterm birth, cesarean section, birth weight, congenital malformations, stillbirth) is more limited.^8^^,^^9^^,^^12^^,^^13^ Another Swedish population-based study^8^ reported increased risks for adverse outcomes in women with MASLD, but sample size was limited (110 MASLD pregnancies), the study period (1992–2011) does not reflect the most recent advances in clinical practice, and the authors were not able to assess adverse outcomes by disease severity. A large study^9^ (5640 pregnancies) based on weighted discharge data from the United States (US) national inpatient sample (2007–2016) also reported increased adverse outcomes such as hypertensive complications, and preterm birth. However, specificity of MASLD diagnosis was potentially low due to their International Classification of Diseases (ICD)-based MASLD definition. Other previous studies were small (<137 pregnancies in each study)^7^^,^^10^^,^^11^^,^^14^, ^15^, ^16^, ^17^ and restricted to data from single healthcare centers which may not be representative of the general population of pregnant individuals with MASLD.^7^^,^^10^, ^11^, ^12^^,^^14^, ^15^, ^16^, ^17^ A common limitation among all previous studies is that they did not evaluate if intrafamilial factors, such as environmental and genetic factors, play a role for pregnancy outcome. Prior studies also lacked information on the histological subgroups of MASLD which range from simple steatosis to the inflammatory state of steatohepatitis (metabolic dysfunction-associated steatohepatitis, MASH), which may further progress to noncirrhotic fibrosis, and cirrhosis.^2^^,^^18^

We aimed to perform a nationwide matched cohort study comparing adverse pregnancy and birth outcomes in births of women with biopsy-proven MASLD compared with matched reference births of women without MASLD. We aimed to assess adverse outcomes overall, by histological subgroups, and to evaluate the role of overweight and obesity.

## Methods

### Data sources and study design

We performed a nationwide matched cohort study based on the ESPRESSO (Epidemiology Strengthened by histopathology Reports in Sweden)^19^ cohort between 1992 and 2017. We obtained liver biopsy data from all 28 pathology departments in Sweden and linked them to nationwide Swedish healthcare registers.^19^^,^^20^ We performed linkage using the unique personal identity number assigned to all permanent Swedish residents at time of birth or immigration.^20^ We used the *Medical Birth Register* (*MBR*), which started in 1973, and covers 99% of all births in Sweden. It contains high-quality data on pregnancy and birth outcomes, as well as on confounders such as body mass index (BMI) and smoking (recorded since 1992 and 1982 respectively).^21^ The high validity and completeness of the *MBR* is owed to Sweden's universal access to antenatal care and the semi-automated extraction of data from the regional electronic health records.^21^ Data are prospectively collected from the beginning of the first prenatal visit which usually takes place before the 12th gestational week.^21^ We obtained data on relevant comorbidities from the *National Patient Register (NPR)* which, since 1964, stores data on diagnoses and procedures in non-primary healthcare (positive predictive value [PPV] = 85–95% for most diagnoses).^22^ The *NPR* became nationwide in 1987 and added data on (non-primary) outpatient visits in 2001.^22^ We used the *Prescribed Drug Register (PDR)* to define certain comorbidities. This register contains information on medications dispensed at Swedish pharmacies since July 1, 2005.^23^ We extracted data on education from the Swedish *Longitudinal Integrated Database for Health Insurance and Labour Market Studies* (*LISA; PPV* = *85%*),^24^ retrieved sibling information from the *Total Population Register* (i.e., complete linkage to all siblings with residence in Sweden since date of birth or immigration),^25^ and extracted data on deaths from the *Cause of Death Register* which covers all deaths in Sweden since 1952.^26^

### MASLD

#### Inclusion and exclusion criteria

We identified MASLD through the first liver biopsy histopathology report that included a SNOMED topography code for liver (T56), and a SNOMED morphology code for steatosis (M5008× or M5520×) between 01/01/1965 and 31/12/2017 (index liver biopsy). To further enhance the specificity of our identification of MASLD, we excluded 1) individuals with a recorded ICD code for other concomitant chronic liver conditions prior to the delivery date such as prior alcohol abuse/misuse, recorded other etiology of acute or chronic liver disease, or liver transplantation (Table S1) and 2) individuals who emigrated from Sweden prior to the index liver biopsy date. This ICD-based algorithm follows international expert panel consensus recommendations,^27^ and was previously validated (PPV = 92%).^28^ Recently, it was confirmed that according to this registry-based definition of MASLD based on Swedish health registers, more than 99.5% of patients with NAFLD meet the new MASLD criteria.^29^ A two-stage Delphi process after the name change demonstrated that ICD codes for NAFLD can be used to classify MASLD.^30^ The date of the first MASLD diagnosis was defined as the date of the index liver biopsy and was required to be prior to the date of delivery.

#### Histological subgroups

We identified histological subgroups of MASLD using SNOMED definitions^31^ for coherent nationwide histopathology reporting in Sweden and based on the most recent histopathology report with MASLD before the date of delivery. Histological subgroups included simple steatosis, MASH without fibrosis, noncirrhotic fibrosis, or cirrhosis (definitions in Table S2).^28^ In stratified analyses, we pooled all individuals with MASH with or without fibrosis and cirrhosis (severe MASLD).

### Births of women with MASLD

We identified singleton births of women with MASLD aged 15–44 years at delivery through the *MBR*.

### Reference births of women and female siblings without MASLD

We used singleton births of reference women without diagnosed MASLD (i.e., without ICD code K76) and without any other liver disease (definitions in Table S1) aged 15–44 years at delivery as comparators. In a sibling-controlled analysis, we identified all singleton births in female full siblings (sisters) without diagnosed MASLD (i.e., without ICD code K76) to women with MASLD.

### Matching of births

**Fig. 1:**
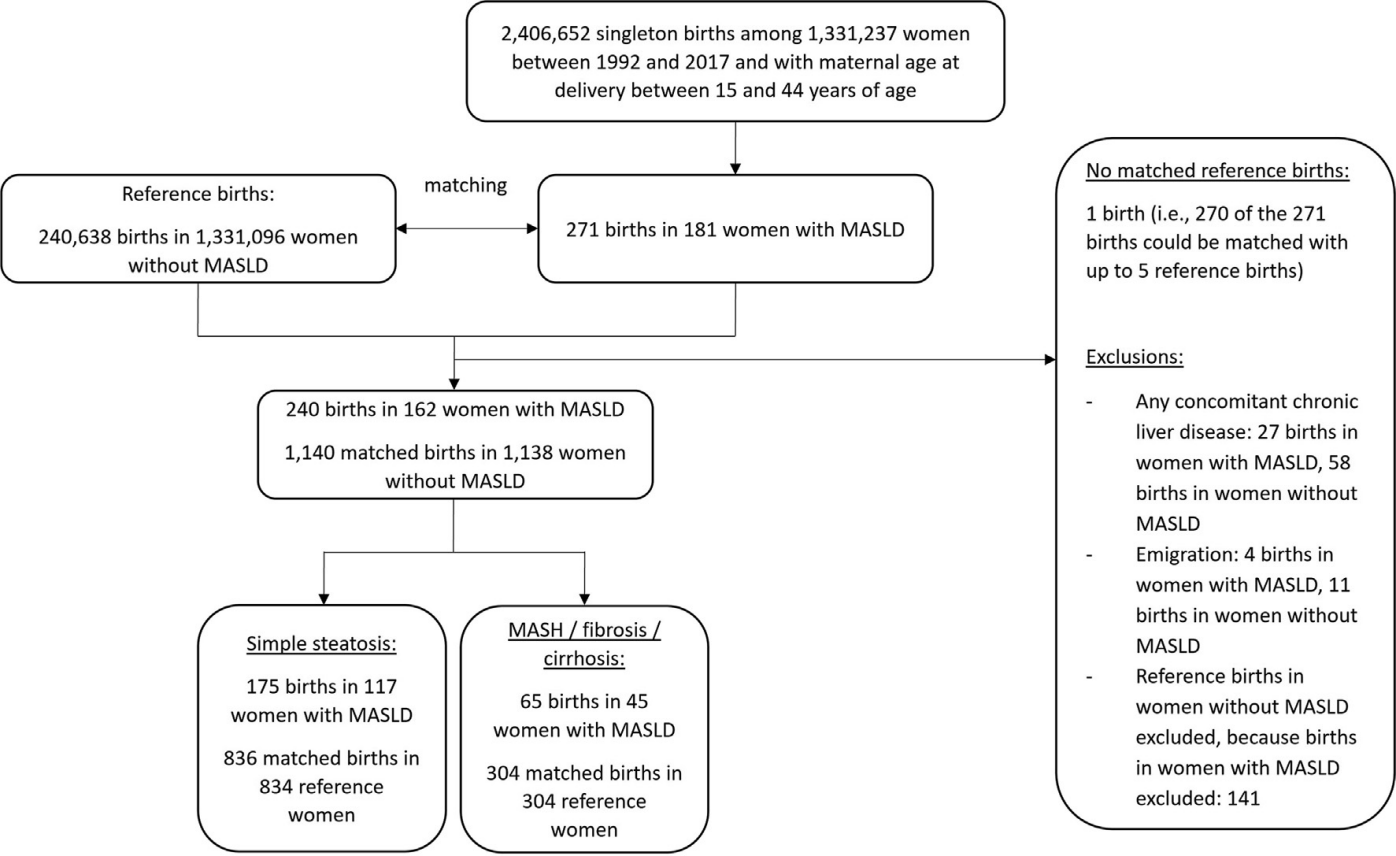
Flow chart of cohort enrolment. Abbreviations: MASLD, metabolic dysfunction-associated steatotic liver disease; MASH, metabolic dysfunction-associated steatohepatitis.

The observation unit was a singleton birth, but women could contribute several births to the study population. We matched each singleton birth of a woman with MASLD with up to five singleton births of reference women according to maternal age at delivery, calendar year of delivery, and parity (Fig. 1).

### Outcomes

The primary outcome was preterm birth, divided into any preterm birth (<37 gestational weeks) and very preterm birth (<32 gestational weeks). We further distinguished between medically indicated and spontaneous preterm birth. The secondary outcomes were small for gestational age (SGA, birth weight <10th percentile below the sex-specific mean weight for gestational age), large for gestational age (LGA, birth weight >90th percentile above the sex-specific mean weight for gestational age), low birth weight (<2500 g; analyzed separately for all deliveries and full-term deliveries), Apgar <7 at 5 min in full-term deliveries, congenital malformations at birth and up until one year after birth, neonatal death (death within 28 days after delivery), stillbirth (death after 28 gestational weeks until 07/2008 and thereafter after 22 gestational weeks), induction of labor, cesarean section (emergency vs. elective), and instrumental delivery (definitions: Table S3). *Post-hoc*, we evaluated in the main analysis the outcomes pre-eclampsia and macrosomia (birth weight >4000 g; Table S3). Macrosomia was analyzed separately for all deliveries and full-term deliveries.

### Statistical analyses

We used multivariable conditional logistic regression and presented odds ratios (ORs) with 95% confidence intervals (CIs). For each risk estimate we presented ORs (1) conditioned on the matching set including maternal age at delivery, calendar year of delivery, and parity (model 1), and (2) additionally adjusted for level of education, country of birth, BMI in early pregnancy, smoking in early pregnancy, as well as diabetes (type 1, type 2, and gestational diabetes), hypertension (including gestational hypertension), dyslipidemia, and pre-eclampsia prior to delivery (model 2; covariate definitions in Table S4). The ORs reported in the manuscript are those additionally adjusted in model 2, except for 1) neonatal death and stillbirth, for which we report the ORs from model 1 due to few events, and 2) sibling analyses where we report ORs from model 2 with fewer adjustments due to few outcomes. This restricted model 2 was conditioned on the matching set and additionally adjusted for BMI in early pregnancy, as well as diabetes and hypertension prior to delivery. We included education, BMI, and smoking as categorical covariates with missing indicator in the models.

We examined stillbirth, induction of labor, cesarean section, and instrumental delivery in all births, while the remaining outcomes were examined in live births only. Logistic regression was also used for neonatal death given short follow-up time (28-day period after delivery).

In pre-specified sub-analyses, we examined the outcomes by histological subgroups of MASLD, namely simple steatosis vs. severe MASLD. We reported adjusted ORs (aOR) from model 2 for any preterm birth, cesarean section, birth weight parameters, and induction of labor. For the other outcomes, we do not report results as the number of events were small resulting in unstable aORs.

We carried out the following pre-specified sensitivity analyses. First, to reduce shared intrafamilial confounding, we repeated analyses using births in female full sibling comparators (sisters). Second, we restricted reference births to women *without* obesity (BMI <30 kg/m^2^) due to the likelihood that obese women also have (undetected) MASLD. Third, we instead restricted births to women *with* overweight or obesity (BMI ≥25 kg/m^2^) to better understand the exclusive role of MASLD. Fourth, we stratified by parity. We conducted sensitivity analyses exclusively for preterm birth and cesarean section.

#### Post-hoc sensitivity analyses

First, we investigated the effect of maternal disease duration by estimating aOR for preterm birth and cesarean section in births of women with a disease duration of <5 vs. ≥5 years before delivery.

Second, we changed the considered time period of metabolic-related covariates (diabetes, hypertension, pre-eclampsia, and dyslipidemia) to a) any time prior to the index liver biopsy (i.e., first biopsy indicating MASLD; model 3) and b) any time prior to start of pregnancy (model 4) to study if metabolic disorders lay on the causal pathway between maternal MASLD and maternal–fetal outcomes.

Third, to further explore if pregnancy-specific metabolic disorders during the respective pregnancy are mediating the association between maternal MASLD and maternal–fetal outcomes, we restricted the study population to births of women without a) gestational diabetes, b) gestational hypertension, and c) pre-eclampsia.

All statistical analyses were performed using R version 4.3 (R Foundation for Statistical Computing, Vienna, Austria).

### Ethical approval

This study was approved by the Ethics Review Board in Stockholm, Sweden (2014/1287-31/4, 2018/972-32 and 2022-05774-02).

### Informed consent statement

Patient consent was waived due to the fact that researchers only received anonymized data.

### Role of the funding source

C.A.M. was supported by the Swiss National Science Foundation (P500PM_217670). F.E. was supported by the Swiss National Science Foundation (P500PM_210866). J.S. was supported by European Crohn's and Colitis Organisation and The Swedish Society for Medical Research (PG-23-0315-H-02). J.F.L. was supported by Karolinska Institutet.

## Results

### Study cohort

Our final cohort included 240 singleton births to 162 women with MASLD matched to 1140 comparator births to 1138 reference women (Fig. 1). Around 68% of women with MASLD were diagnosed before 1999 and the median time between MASLD diagnosis and delivery was 5.6 years (IQR 3.2–9.9). Most deliveries were between 2000 and 2010 (52% in births of women with MASLD and in reference births). The median maternal age at delivery was 32 years (IQR 27–36) and 39% of pregnancies were nulliparous. The proportion of women born in a non-Nordic country was similar in those with (15%) vs. without known MASLD (17%), but women with MASLD had lower levels of education (27% vs. 45% with ≥13 years of education). Women with MASLD, compared to reference women, smoked more frequently during early pregnancy (17% vs. 10%), were more frequently obese (38% vs. 10%), and had higher rates of prior diabetes (10.4% vs. 0.9%), hypertension (5.0% vs. 0.5%), dyslipidemia (1.7% vs. 0.2%), and pre-eclampsia (6.2% vs. 2.7%, Table 1).

**Table 1:** Baseline characteristics of births in women with MASLD and matched births in reference women without known MASLD.

	Births in women with MASLD (n births = 240)	Births in reference women (n births = 1140)
**Women, n**	162	1138
**Maternal age at delivery [years]**		
Mean (SD)	31.5 (5.6)	31.5 (5.6)
Median (IQR)	32 (27–36)	32 (27–36)
Range, min–max	18–43	18–43
15–<25	26 (11)	123 (11)
25–<35	135 (56)	643 (56)
35–44	79 (33)	374 (33)
**Pregnancy duration [days]**		
Mean (SD)	271.2 (18.1)	278.7 (13.9)
Median (IQR)	274 (264–283)	281 (274–287)
Range, min–max	188–305	163–301
**Calendar year of delivery**		
1992–1999	65 (27)	314 (28)
2000–2010	125 (52)	595 (52)
2011–2017	50 (21)	231 (20)
**Histological subgroup of MASLD**		
Simple steatosis	175 (73)	–
MASH without fibrosis	30 (13)	–
Noncirrhotic fibrosis	31 (13)	–
Cirrhosis	4 (2)	–
**Year of first MASLD diagnosis (index liver biopsy)**		
Before 1999	162 (68)	–
2000–2010	71 (30)	–
2011–2017	7 (3)	–
**Disease duration (time between first MASLD diagnosis and delivery) [years]**		
Mean (SD)	7.0 (5.0)	–
Median (IQR)	5.6 (3.2–9.9)	–
Range, min–max	0.0–26.5	–
<5	102 (42)	–
5–10	81 (34)	–
≥10	57 (24)	–
**Maternal country of birth**		
Nordic	203 (85)	945 (83)
Other	37 (15)	195 (17)
**Civil status of the mother**		
Living with partner	207 (86)	1027 (90)
Not living with partner	12 (5)	21 (2)
Missing	21 (9)	92 (8)
**Level of education**		
Compulsory school (≤9 years)	36 (15)	117 (10)
Upper secondary school (10–12 years)	140 (58)	489 (43)
College or university (≥13 years)	64 (27)	512 (45)
Missing	0 (0)	22 (2)
**Parity**		
Nulliparous	93 (39)	442 (39)
Multiparous	147 (61)	698 (61)
**BMI in early pregnancy [kg/m^2^]**		
Mean (SD)	29.3 (5.8)	24.7 (4.6)
Median (IQR)	28.7 (25.0–33.2)	23.9 (21.5–26.8)
Range, min–max	18.9–48.2	17.3–61.3
<18.5	0 (0)	24 (2)
18.5–<25	55 (23)	591 (52)
25–<30	72 (30)	272 (24)
≥30	92 (38)	117 (10)
Missing	21 (9)	136 (12)
**Smoking in early pregnancy**		
No	187 (78)	967 (85)
Yes	41 (17)	115 (10)
Missing	12 (5)	58 (5)
**Prior comorbidities and conditions**		
Diabetes (including gestational diabetes)	25 (10.4)	10 (0.9)
Hypertension (including gestational hypertension)	12 (5.0)	6 (0.5)
Dyslipidemia	4 (1.7)	2 (0.2)
Pre-eclampsia	15 (6.2)	31 (2.7)

Note: Data are presented as number (%) except where indicated otherwise.

Abbreviations: MASLD, metabolic dysfunction-associated steatotic liver disease; n, number; SD, standard deviation; IQR, interquartile range; min, minimum, max, maximum; BMI, body mass index.

In women with MASLD, 73% (n = 175) had liver histopathology indicating simple steatosis alone, while 27% (n = 65) had severe MASLD (Table 1, description of characteristics by MASLD severity in Table S5). Among those with severe MASLD, 30 women (13%) had MASH without fibrosis, 31 women (13%) had noncirrhotic fibrosis, and four women (2%) had liver cirrhosis (Table 1).

### Preterm birth

**Fig. 2:**
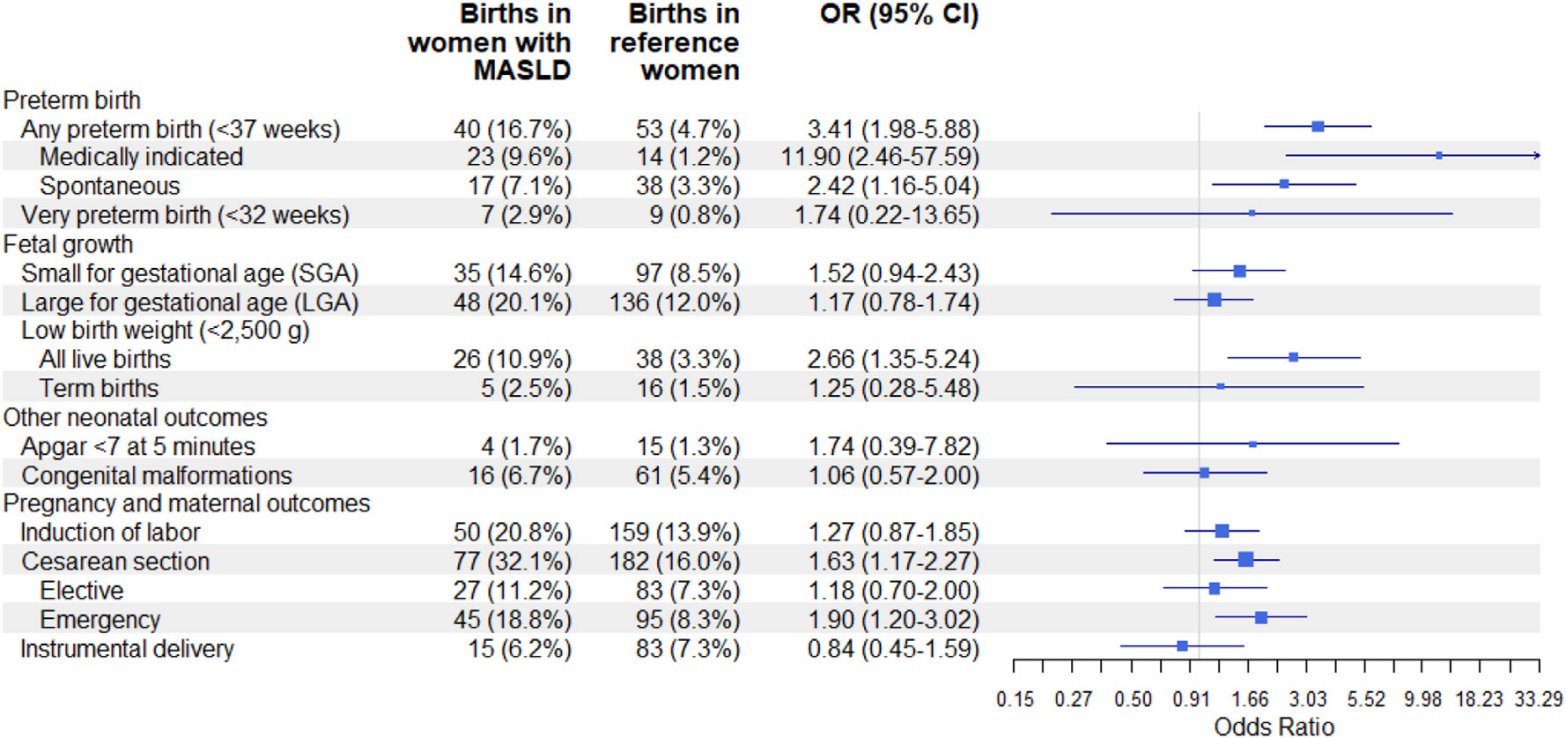
Forest plot of pregnancy and birth outcomes for all births in women with MASLD vs. births in reference women without known MASLD.

In our study population, 93 preterm births occurred, of which 40 (16.7%) occurred in women with MASLD compared to 53 (4.7%) in reference births, yielding an aOR of 3.41 (95% CI = 1.98–5.88; Table 2, Fig. 2: Forest plot). The association was observed for both medically indicated preterm birth (OR 11.90, 95% CI 2.46–57.59; 37 events in total) and spontaneous preterm birth (aOR 2.42, 95% CI 1.16–5.04; 55 events in total). The absolute numbers of very preterm births were low (16 events in total), of which seven events (2.9%) occurred in women with MASLD and nine events (0.8%) in reference women. This resulted in no association between MASLD and very preterm birth, but the CI was wide (aOR of 1.74, 95% CI 0.22–13.65).

**Table 2:** Pregnancy and birth outcomes for all births in women with MASLD vs. births in reference women without known MASLD.

	Births in women with MASLD n (%)	Births in reference women n (%)	OR^a^ (95% CI)	OR^b^ (95% CI)
Live births, n	239	1136		
Preterm birth				
Any preterm birth (<37 weeks)	40 (16.7%)	53 (4.7%)	4.30 (2.74–6.74)	3.41 (1.98–5.88)
Medically indicated	23 (9.6%)	14 (1.2%)	7.88 (4.05–15.33)	11.90 (2.46–57.59)
Spontaneous	17 (7.1%)	38 (3.3%)	2.29 (1.26–4.15)	2.42 (1.16–5.04)
Very preterm birth (<32 weeks)	7 (2.9%)	9 (0.8%)	3.94 (1.42–10.94)	1.74 (0.22–13.65)
Pre-eclampsia	15 (6.2%)	31 (2.7%)	2.29 (1.23–4.30)	1.48 (0.65–3.37)
Birth weight parameters				
Small for gestational age (SGA)	35 (14.6%)	97 (8.5%)	1.84 (1.21–2.79)	1.52 (0.94–2.43)
Large for gestational age (LGA)	48 (20.1%)	136 (12.0%)	1.84 (1.27–2.67)	1.17 (0.78–1.74)
Low birth weight (<2500 g)				
All live births	26 (10.9%)	38 (3.3%)	3.92 (2.25–6.83)	2.66 (1.35–5.24)
Term births	5 (2.5%)	16 (1.5%)	2.55 (0.85–7.62)	1.25 (0.28–5.48)
Missing information	1 (0.4%)	4 (0.4%)		
Macrosomia (>4000 g)				
All live births	46 (19.2%)	233 (20.5%)	0.90 (0.62–1.29)	0.72 (0.50–1.04)
Term births	44 (22.1%)	233 (21.5%)	1.01 (0.69–1.49)	0.79 (0.55–1.15)
Other neonatal outcomes				
Apgar <7 at 5 min	4 (1.7%)	15 (1.3%)	1.25 (0.42–3.78)	1.74 (0.39–7.82)
Missing information	2 (0.8%)	9 (0.8%)		
Congenital malformations	16 (6.7%)	61 (5.4%)	1.26 (0.71–2.26)	1.06 (0.57–2.00)
Missing information	1 (0.4%)	4 (0.4%)		
Neonatal death	1 (0.4%)	2 (0.2%)	2.50 (0.23–27.57)	NE
All births, n	240	1140		
Intrauterine fetal death				
Stillbirth	1 (0.4%)	4 (0.4%)	1.11 (0.12–10.06)	NE
Pregnancy and maternal outcomes				
Induction of labor	50 (20.8%)	159 (13.9%)	1.63 (1.14–2.32)	1.27 (0.87–1.85)
Cesarean section	77 (32.1%)	182 (16.0%)	2.70 (1.93–3.78)	1.63 (1.17–2.27)
Elective	27 (11.2%)	83 (7.3%)	1.69 (1.05–2.72)	1.18 (0.70–2.00)
Emergency	45 (18.8%)	95 (8.3%)	2.78 (1.85–4.18)	1.90 (1.20–3.02)
Instrumental delivery	15 (6.2%)	83 (7.3%)	0.83 (0.46–1.50)	0.84 (0.45–1.59)

Abbreviations: MASLD, metabolic dysfunction-associated steatotic liver disease; n, number; OR, odds ratio; CI, confidence interval; NE, not estimable.

a Model 1: Conditioned on the matching set (maternal age at delivery, calendar year of delivery, and parity).

b Model 2: Conditioned on the matching set and additionally adjusted for level of education, country of birth, BMI in early pregnancy, smoking in early pregnancy, and the following conditions prior to delivery: diabetes (type 1, type 2, and gestational diabetes), hypertension (including gestational hypertension), dyslipidemia, and pre-eclampsia.

In women with simple steatosis alone, 31 preterm births occurred (17.7% vs. 38 [4.6%] in reference births), yielding an aOR of 4.30 (95% CI 2.32–7.96, Table S6). In women with severe MASLD, 9 preterm births occurred (14.1% vs. 15 [5.0%] in reference births), yielding an aOR of 1.53 (95% CI 0.23–10.02; Table S7).

The risk of any preterm birth remained increased in the sibling analysis (aOR 18.92, 95% CI 3.83–93.54, Table S9)—which included 78 births to 45 women with MASLD and their siblings without known MASLD (Table S8)—and remained increased in all other sensitivity analyses (Tables S10–S13).

### Other fetal outcomes

We found no association with SGA (aOR 1.52, 95% CI 0.94–2.43; 132 events in total) and LGA (aOR 1.17, 95% CI 0.78–1.74; 184 events in total), but the aORs in women with severe MASLD were higher compared to women with simple steatosis (1.73 vs. 1.60 [SGA] respectively 1.43 vs. 1.06 [LGA]). We found an association with low birth weight (aOR 2.66, 95% CI 1.35–5.24; 64 events in total; Table 2, Fig. 2), but when restricting to term births (≥37 weeks), the association vanished (aOR 1.25, 95% CI 0.28–5.48; 21 events in total). The *post-hoc* inclusion of macrosomia (birth weight >4000 g) as outcome has shown no association (all live births: aOR 0.72, 95% CI 0.50–1.04, term births: 0.79, 95% CI 0.55–1.15). We observed no association with congenital malformations (aOR 1.06, 95% CI 0.57–2.00; 77 events in total), or Apgar <7 at 5 min (aOR 1.74, 95% CI 0.39–7.82; 19 events in total), but absolute numbers for the latter were small both in women with MASLD (n = 15 [1.3%]) and reference women (n = 4 [1.7%]).

### Stillbirth and neonatal death

We observed three neonatal deaths in total, of which one (0.4%) was in women with MASLD and two (0.2%) in reference women. We further observed five stillbirths in total, of which one (0.4%) was in a woman with MASLD and four (0.4%) in reference women (Table 2). For both outcomes, this resulted in no increased risk based on ORs from model 1 (Table 2, Fig. 2).

### Pregnancy and maternal outcomes

#### Cesarean section

In our study population, 259 cesarean sections occurred, of which 77 (32.1%) occurred in births of women with MASLD compared to 182 (13.9%) in reference births, yielding an aOR of 1.63 (95% CI 1.17–2.27). The increased risk was driven by in total 140 emergency cesarean sections (aOR 1.90, 95% CI 1.20–3.02). We observed no association with elective cesarean section (aOR 1.18, 95% CI 0.70–2.00; Table 2, Fig. 2), which occurred in 110 births in total.

The odds of emergency cesarean section remained increased in women with simple steatosis alone (aOR 2.17, 95% CI 1.30–3.65; 56 events [32.0%] vs. 130 [15.6%] in reference births; Table S6) but not in those with severe MASLD (aOR 0.95, 95% CI 0.27–3.40; 21 events [32.3%] vs. 52 events [17.1%] in reference births; Tables S7). In contrast, the aOR of elective cesarean section was higher in women with severe MASLD compared to women with simple steatosis alone (1.93 vs. 0.94; Tables S6 and S7).

The odds of cesarean section remained increased in the sibling analyses (aOR 1.94, 95% CI 1.02–3.71, Table S9), and in further sensitivity analyses (Tables S10, S12 and S13), but not when restricting to reference women with obesity (aOR 1.20, 95% CI 0.77–1.86, Table S11). The latter sensitivity analysis also did not confirm the increased risk of emergency cesarean section observed in the main analysis (aOR 1.35, 95% CI 0.67–2.75, Table S11), while all other sensitivity analyses did (Tables S9 and S10, S12 and S13). Lastly, the odds of elective cesarean section remained unchanged across all sensitivity analyses, consistent with the findings of the main analysis (i.e., no difference between the two groups).

#### Other pregnancy and maternal outcomes

We observed no association with instrumental delivery (aOR 0.84, 95% CI 0.45–1.59; 98 events in total; Table 2, Fig. 2) or induction of labor (aOR 1.27, 95% CI 0.87–1.85; 209 events in total), but for the latter we found higher aOR among women with severe MASLD compared to women with simple steatosis (1.83 vs. 1.13; Tables S6 and S7). In a *post-hoc* analysis, we found no association with pre-eclampsia (aOR 1.48, 95% CI 0.65–3.37, 46 events in total).

#### Effect of maternal disease duration before delivery

The risk of any preterm birth and cesarean section remained increased when investigating births of mothers with a disease duration of <5 years vs. ≥5 years before delivery (Table S14).

#### Changing time windows of considered metabolic-related covariates

When changing the considered time period of metabolic-related covariates (diabetes, hypertension, pre-eclampsia, and dyslipidemia) from any time prior to delivery to a) any time prior to the index liver biopsy (i.e., first liver biopsy indicating MASLD) and b) to any time prior to start of pregnancy, the results did not meaningfully change (Table S15). However, in case of any preterm birth, the aOR increased from 3.41 (95% CI 1.98–5.88) in the main analysis to a) 4.11 (95% CI 2.39–7.05) respectively b) 4.00 (95% CI 2.31–6.93; Table S15) in the sensitivity analyses, but 95% CIs were overlapping.

#### Restriction to births of pregnancies without pregnancy-specific metabolic disorders

Our results did not change when restricting our study population to births of women without a) gestational diabetes (Table S17), b) gestational hypertension (Table S18), or c) pre-eclampsia (Table S19).

## Discussion

This nationwide cohort study investigating pregnancy and birth outcomes in women with biopsy-proven MASLD compared with matched reference women without known MASLD had five key findings: First, MASLD was associated with a higher risk of preterm birth, independent of obesity or intrafamilial factors. Second, women with MASLD were more likely to have cesarean section, however this observation was most likely explained by a higher prevalence of obesity. Third, MASLD did not affect birth weight parameters. Fourth, the small number of congenital malformations, stillbirths, and neonatal deaths among MASLD women is reassuring, even if CIs were wide when comparing to reference births. Finally, we did not find higher risks for adverse outcomes in women with evidence of moresevere MASLD, but statistical power was limited.

Our results clearly show that, independent of obesity and parity, MASLD strongly increases the risk of preterm birth which does not appear to be attributable to intrafamilial factors as the estimates remained when compared to siblings; however, CIs were wide. As we adjusted for a range of other potential confounders including gestational diabetes,^32^ and performed several sensitivity analyses (including evaluating if gestational hypertension, gestational diabetes, or pre-eclampsia lie on the causal pathway), this suggests that MASLD might be an independent risk factor for preterm birth. The mechanism behind this relationship remains to be elucidated. Reassuringly, the absolute number of very preterm birth, with even worse adverse effects on motor and psychological development across the life span than any preterm birth,^33^ was low, and resulted in no association between MASLD and very preterm birth. However, statistical power was limited. Our reported 3.4-fold increased risk of preterm birth is higher compared to the 2.5-fold increased risk described in another Swedish observational study (1992–2011) that included 110 pregnancies in women with MASLD identified by ICD-codes,^8^ and compared to the 1.6-fold increased risk reported by the largest study evaluating pregnancy and birth outcomes in 5640 MASLD women (identified by ICD-codes) based on the weighted discharge data from the US national inpatient sample (2007–2016).^9^ These differences may be explained by the higher specificity of MASLD diagnosis in our study compared to the Swedish and US study potentially leading to an overrepresentation of more severe cases resulting in higher rates of adverse outcomes (although in our study, the offspring of women with severe MASLD were no worse off than the offspring of women with simple steatosis, but statistical power was limited). Reassuringly, our data allowed to distinguish between medically indicated and spontaneous preterm birth allowing for more differentiated implications for clinical practice and further research. Overall, MASLD was associated with both, spontaneous and medically indicated preterm birth, but driven primarily, but not only, by medically indicated preterm birth. The increased risk of medically indicated preterm birth further suggests that MASLD leads to complications necessitating medical intervention such as cesarean section. It is expected that with MASLD associated metabolic disorders such as (gestational) diabetes and hypertension, pre-eclampsia, dyslipidemia, and obesity play a major role.^7^, ^8^, ^9^, ^10^^,^^14^^,^^16^ These conditions were, as expected, more common in pregnant women with MASLD compared to reference women, but since we adjusted for those confounding factors and performed sensitivity analyses by restricting to different BMI strata, pregnancies of women with MASLD seem to lead to more complications towards the delivery date leading to more medically indicated preterm births. A large analysis of hospital discharge records from the US national inpatient sample (2009–2019) reported more cardiovascular complications during delivery hospitalizations in 17,593 MASLD women, which might contribute to higher rates of medically indicated preterm births in our study compared to reference women.^34^ Further, we cannot rule out that lower socioeconomic status in women with MASLD played a role despite adjustments for level of education (proxy for socioeconomic status). In contrast to previous studies,^8^^,^^9^ our data allowed us to assess preterm birth by MASLD severity. Preterm birth did not increase with disease severity. This lack of positive association may be attributed to the small sample size resulting in low statistical power: MASLD is a progressive disease and increasing disease severity potentially reduces fertility. This led to only 65 births among women with severe MASLD (27% of our study population). It is possible that women with severe MASLD receive closer monitoring in clinical practice, resulting in better care and consequently fewer medically indicated preterm births compared to women with simple steatosis alone. However, a large study based on discharge data from the US National Inpatient Sample (2012–2016) including 18,573,000 deliveries of women with cirrhosis supports the notion that the risk of preterm birth increases with MASLD severity^35^: The study reported a 2-fold increased risk of preterm birth (95% CI 1.3–3.3) among women with cirrhosis compared to non-cirrhotic chronic liver disease.^35^

Our results suggest that, independent of parity, MASLD increases the risk of cesarean section, which is likely not due to familial factors as the estimates remained when compared to siblings, and likely not due to gestational diabetes, gestational hypertension, or pre-eclampsia as results remained consistent when restricting to women without those conditions. However, the association might be explained by obesity as the association vanished after restricting to reference births in women who were overweight or obese. This suggests that MASLD is not an independent risk factor of cesarean section, which is supported by a Chinese hospital-based study (2013–2020).^12^ The Chinese study showed that MASLD and cesarean section are associated in those with a normal BMI, but not in overweight or obese pregnant women.^12^ However, we cannot completely rule out the possibility that our finding of a risk increase for cesarean section occurred by chance. It is possible that adjustment for BMI in the main analysis did not fully take out the impact of BMI resulting in a decreased estimate when restricting to overweight or obese women. The previous Swedish study by Hagström et al. described similar risk estimates (1.5-fold increased risk) but did not further explore the role of obesity on cesarean section.^8^ In addition, our study was able to distinguish between emergency and elective cesarean sections providing further evidence for clinical practice. It appears that the 1.6-fold higher risk of cesarean section in women with MASLD is due to emergency cesarean sections (no increased risk of elective cesarean sections). Complications towards delivery resulting in emergency cesarean sections might be due to overweight or obesity as the association vanished when restricting to births of women with overweight/obesity, but future studies should evaluate if fetuses of mothers with MASLD were more likely to show distress antenatally or in labor as possible reasons for emergency cesarean section. Additionally, our results suggest that clinical practices related to MASLD severity may further influence the type of cesarean section. Interestingly, simple steatosis alone was associated with emergency cesarean section, while severe MASLD tended to have more elective than emergency cesarean sections. It is possible that obstetricians in Sweden do not consider patients with simple steatosis alone as having high-risk pregnancies, resulting in less monitoring compared to women with severe MASLD. This may lead to a higher incidence of emergency cesarean sections in those with simple steatosis alone.

Further, we did not find evidence that MASLD impacts birth weight parameters which is in line with previous literature.^8^^,^^9^^,^^12^ This included the large US study which studied fetal growth restriction and LGA (both defined by ICD codes),^9^ the hospital-based study from China evaluating low birth weight,^12^ and the Swedish study which assessed the association with SGA and low birth weight.^8^ The latter two found an increased risk of low birth weight before adjusting for gestational age^12^ respectively by including preterm births,^8^ which underlines the importance of assessing low birth weight in only term deliveries. However, we cannot fully rule out that MASLD influences birth weight parameters. It is possible that some mechanisms push towards SGA^36^ while others push towards LGA.^37^ In addition, due to insufficient power, we cannot conclusively assess if greater disease severity negatively impacts birth weight parameters.

In our study, MASLD did not increase the risk of pre-eclampsia, which contrasts with previous studies (e.g., Hagström et al.^8^). It is possible that our study was underpowered in detecting an association. Our results suggest that MASLD does not increase the risk of congenital malformations, despite the potential overrepresentation of more severe cases due to our biopsy-proven MASLD definition. This finding corroborates the results of the other Swedish study, which was based on a smaller sample size and lower sensitivity of MASLD diagnoses.^8^ However, we cannot rule out that MASLD leads to early terminations of pregnancy due to severe fetal anomalies. As we studied births and not conceptions, we were unable to investigate this further.

We were not able to adjust for all confounders when assessing the risk of stillbirth and neonatal death due to the low absolute number of events. However, we suggest that MASLD likely does not increase the risk of stillbirth and neonatal death even though we potentially overrepresent more severe cases in our study. The absolute numbers of stillbirths were equal in both the MASLD and non-MASLD groups (0.4%), which falls within the range of stillbirth prevalence in Sweden (0.35%–0.40%^38^), and results were similar in the smaller Swedish study.^8^ The absolute numbers of neonatal deaths were slightly higher in births of women with MASLD (0.4%, one neonatal death) compared to the background risk in Sweden (0.13–0.18%^38^) and compared to the smaller Swedish study (no neonatal death). However, given the relatively small study population, the single neonatal death was not deemed alarming.

Strengths of this study include its population-based nature, encompassing almost all (99%) births in Sweden. Our study is larger than most previous studies, with a high specificity of MASLD diagnosis based on liver histology (PPV = 92%) and a high validity of other covariates (PPV = 85–95% for most diagnoses in the Patient Register^22^; estimated accuracy of education data: 85%^24^). Prior studies have been limited primarily by reliance on diagnostic codes to identify women with MASLD, potentially resulting in under-reporting and misclassification of MASLD in pregnant women. This is the only study so far which was able to compare the risk magnitude in simple steatosis alone vs. severe MASLD, as well as to determine if associations can be explained by familial factors. Lastly, we had comprehensive data on BMI and smoking, allowing for adjustment of these potential confounders and the performance of sensitivity analyses by BMI strata.

The following limitations need to be considered. First, we cannot rule out residual confounding as data on more granular parameters such as alcohol consumption, laboratory results, and severity of comorbidities were not systematically obtained. Second, several studies have demonstrated strong variability across ethnicities in susceptibility to MASLD, but Swedish registers do not collect information on ethnicity. Thus, since Sweden has a predominantly Caucasian population, our findings may not be generalizable to other ethnicities. Third, despite the larger sample size compared to most studies, we could not perform sub-analyses and sensitivity analyses for all outcomes. Fourth, despite some degree of selection bias inherent in all studies with biopsy-proven MASLD, we believe that our results may be relevant to most women with diagnosed MASLD.^39^ Further, we urge caution when interpreting our risk estimates since power was limited, and the large number of comparisons may result in type 1 error with false-positive results. Fifth, an alternative comparison could have involved using reference births of women with a liver biopsy that did not indicate MASLD. However, this would introduce relevant bias because in Sweden liver biopsy is primarily recommended to individuals with a suspected relevant liver condition (e.g., autoimmune hepatitis), or suspected significant fibrosis or cirrhosis. On the other hand, including reference births of women without diagnosed MASLD may lead to the inclusion of undetected MASLD cases among women in the reference group, as MASLD is often asymptomatic. This could potentially bias risk estimates towards the null and thus it is possible that our findings underestimated the true risks in pregnant women with MASLD. Sixth, we did not treat separate pregnancies from one woman as entirely independent birth events in our analyses (i.e., a woman could contribute several pregnancies to the study population). Nevertheless, when restricting to the first pregnancy of a woman, our results did not change. Lastly, given that the aOR of preterm birth was higher when only considering metabolic-related covariates before MASLD diagnosis (in addition to other covariates) than when considering metabolic-related covariates any time before delivery (main analysis), we cannot rule out statistical overadjustment in our analyses. However, we conclude that overadjustment was not a major problem, because 1) 95% CIs were overlapping when considering different time windows for covariates, and 2) because this observation was not seen for the other outcomes.

In conclusion, this nationwide study of pregnant women with MASLD confirms that MASLD should be regarded as a high-risk obstetric condition, carrying significant implications for pregnancy care. Importantly, MASLD seems to be a risk factor for preterm birth independent of BMI and intrafamilial factors. A higher rate of cesarean section among women with MASLD was most likely due to obesity. Reassuringly, MASLD was not associated with abnormal birth weight parameters or other severe endpoints, such as neonatal death, or stillbirth. We did not find more adverse outcomes with increasing MASLD severity, but statistical power was limited.

## Contributors

C.A.M.: Conceptualization, funding acquisition, investigation, methodology, project administration, validation, visualization, writing–original draft preparation, writing–review and editing. C.A.M has further accessed and verified the underlying data. F.E.: Conceptualization, investigation, methodology, resources, validation, writing—review & editing. D.B.: Conceptualization, investigation, methodology, validation, writing—review & editing. J.S.: Conceptualization, investigation, methodology, validation, writing—review & editing. H.H.: Conceptualization, investigation, methodology, validation, writing—review & editing. M.T.: Data curation, formal analysis, investigation, methodology, software, validation, writing—review & editing. M.T. has further accessed and verified the underlying data. O.S.: Conceptualization, investigation, methodology, validation, writing—review & editing. J.F.L.: Conceptualization, funding acquisition, investigation, methodology, project administration, resources, supervision, validation, writing—review & editing.

## Data sharing statement

Data are not available because of Swedish data protection regulations.

## Declaration of interests

C.A.M. has no conflict of interest. F.E. has served as an advisory board member for Boehringer Ingelheim. D.B.: has no conflict of interest. J.S.: has no conflict of interest. H.H.: HH:s institutions have received research funding from Astra Zeneca, EchoSens, Gilead, Intercept, MSD, Novo Nordisk and Pfizer. H.H. has served as consultant or on advisory boards for Astra Zeneca, Bristol Myers-Squibb, MSD and Novo Nordisk and has been part of hepatic events adjudication committees for Arrowhead, Boehringer Ingelheim, KOWA and GW Pharma. All disclosures are unrelated to the study. M.T.: has no conflict of interest. O.S.: has no conflict of interest. J.F.L.: Dr Ludvigsson has coordinated an unrelated study on behalf of the Swedish IBD quality register (SWIBREG). That study received funding from Janssen corporation. Dr Ludvigsson has also received financial support from Merck developing a paper reviewing national healthcare registers in China. Dr Ludvigsson has an ongoing research collaboration on celiac disease with Takeda; and additional collaboration within IBD with Merck. All disclosures are unrelated to the study.
