# Supplementary material for: Adverse pregnancy and birth outcomes in women with biopsy-proven MASLD: a nationwide cohort study
Source: eClinicalMedicine. 2025 May 9;83:103238. doi: 10.1016/j.eclinm.2025.103238 (PMC12235392; doi:10.1016/j.eclinm.2025.103238)
Supplement: Supplementary Tables [file mmc1.docx]

**SUPPLEMENTARY MATERIAL**

**Title:** Adverse pregnancy and birth outcomes in women with biopsy-proven MASLD: A nationwide cohort study

**Authors:** Carole A. Marxer, Fahim Ebrahimi, David Bergman, Jiangwei Sun, Hannes Hagström, Marcus Thuresson, Olof Stephansson, Jonas F. Ludvigsson

**Table S1:** Exclusion of any concomitant chronic liver condition among women with MASLD and reference women.

| Excluded conditions^1^ | ICD-8 (1969-1986) | ICD-9 (1987-1996) | ICD-10 (1997-) |
| --- | --- | --- | --- |
| Alcohol abuse / misuse, or Alcohol-related liver disease | 280,00; 281,00; 307,00; 307,10; 307,99; 322; 581,10; 583,10; 261,00; 262,00; 291; 291,1; 303; 571,00; 571,01; 979; 980,00; 980,01; 980,98; 980,99 | 291; 294A; 303; 305A; 357F; 425F; 535D; 571A-D; 760W; 790D; 977D; 980A; 980X; V97B | E24.4; F10; G31.2; G62.1; G72.1; I42.6; K29.2; K70; K85.2; K86.0; Q35.4; R78.0; T51.0; T51.8; T51.9; X65; Y15; Y57.3; Y90; Y91; Z50.2; Z71.4; Z72.1 |
| Other abuse- and drug-related diagnoses | 571,0; E860; N980 | 571A-D | F11-F19 |
| Drug-induced liver disease | − | 573D | K71 |
| Viral hepatitis (e.g. hepatitis B, C) | 070; 999,20 | 070 | B15-19; B00.8; B25.1 |
| Budd-Chiari | − | 453A | I82 |
| Liver abscess | 572 | 572A | K75.0; A06.4 |
| HIV | 079,83; Y40,49; Y41,49 | 279K | B20-B24; F02.4; Z21.9; Z71.7 |
| Hemochromatosis | 273,2 | 275A | E83.1 |
| Wilson’s disease | 273,3 | 275B | E83.0 |
| Autoimmune hepatitis | − | 573D; 571E | K75.4 |
| Primary biliary cholangitis | − | 571G | K74.3; K74.4 |
| Other cholangitis | 574,06 | 576B | K83; K83.0A |
| Alpha-1 antitrypsin deficiency | − | 277G | E88.0 |
| Glycogen storage disease |  | 271W, 271X | E74 |
| Liver transplantation^†^ | − | V42H 5200-5299^†^ | Z94.4 JJC, DJ005; DJ006^†^ |
| Gastric bypass surgery | − | − | JDF^†^ |

Abbreviations: ICD, International Classification of Disease; HIV, human immunodeficiency virus

^†^Liver transplantation and bariatric surgery were further defined via procedure codes.

**^1^** We will exclude any person with a diagnosis for another etiology of liver disease, or alcohol abuse/misuse or alcohol-related liver disease, defined on or prior to the index liver biopsy date.

**Table S2:** Definitions of histological subgroups of MASLD*.

|  | | SNOMED and ICD codes | |
| --- | --- | --- | --- |
| # | **Histological subgroup** | **Inclusion** | **Exclusion** |
| 1 | **Cirrhosis** | M495 [exactly] or M4950x | - |
| 2 | **Noncirrhotic fibrosis (note that this may or may not include MASH)** | Steatosis: either M008x or M5520x, PLUS at least 1 fibrosis code: M49 [exactly], M4900x or M49060. | Cirrhosis codes: M459 [exactly] or M4950x |
| 3 | **MASH without fibrosis** | Steatosis: either M008x or M5520x, PLUS at least one of the following:  1. any M4- code, or 2. M5400x | M4 defines a very broad category of inflammation, both acute and chronic.  Cannot have any of:  Fibrosis codes (M49 [exactly], M4900x or M49060)  OR Cirrhosis: M495 [exactly] or M4950x. |
| 4 | **Simple steatosis** | M5008x or M5520x | Cannot have any:  Inflammation codes: M4- or M5400x  OR Fibrosis: M49 [exactly], M4900x or M49060  OR Cirrhosis: M495 [exactly] or M4950x |

Abbreviations: MASLD, metabolic dysfunction-associated steatotic liver disease; SNOMED, Systematized Nomenclature of Medicine; ICD, International Classification of Diseases.

*Of note: In Sweden, clinically indicated liver biopsies are generally conducted with a single pass of the liver, unless a satisfactory specimen could not be obtained. According to Swedish liver histopathology reporting recommendations, it is documented if any biopsy is too short in length (i.e., <15 mm in length), has fewer than five portal tracts, or is fragmented, respectively (Svensk Förening för Patologi–Svensk Förening för Klinisk Cytologi. Available at: https://www.svfp.se/foreningar/uploads/L15178/kvast/lever/Leverbiopsier2019.pdf. Accessed April 4, 2019).

**Table S3:** Codes for pregnancy and birth outcomes.

| Outcome | Definition | Data source/s |
| --- | --- | --- |
| Preterm birth (any) | <37 gestational weeks | *MBR* |
| Medically indicated preterm birth | - Variables in *MBR:* Induced cesarean section, planned cesarean section - ICD-10: O61 - ICD-9: 659B - Exclusion of women with premature rupture of the membranes (ICD-10: O42, ICD-9: 658B) | *MBR* |
| Spontaneous preterm birth | - Variables in *MBR: Spontaneous preterm birth* - premature rupture of the membranes (ICD-10: O42, ICD-9: 658B) | *MBR* |
| Very preterm birth | <32 gestational weeks | *MBR* |
| Pre-eclampsia | ICD-10: O14-O15  ICD-9: 642E, 642F, 642G, 642H  (recordings during pregnancy of interest; restricted to live births) | *MBR* |
| Small for gestational age (SGA) | Birth weight <10^th^ percentile below the sex specific mean for gestational age according to the Swedish reference curve. | *MBR* |
| Large for gestational age (LGA) | Birth weight >90^th^ percentile above the sex specific mean for gestational age according to the Swedish reference curve. | *MBR* |
| Low birth weight | Birth weight <2,500 g (separate for all live births and term live births [gestational age ≥ week 37+0]) | *MBR* |
| Macrosomia | Birth weight >4,000 g (separate for all live births and term live births [gestational age ≥ week 37+0]) | *MBR* |
| Apgar <7 at 5 minutes | Apgar <7 (restricted to term live births: ≥37 gestational weeks) | *MBR* |
| Congenital malformations | ICD-10: Q00–Q99  ICD-9: 740-759  (recordings within 365 days after delivery; restricted to live births) | *NPR* and *Cause of Death Register* |
| Neonatal death | Death within the first 28 days of delivery | *Cause of Death Register* |
| Stillbirth | Death of fetus after 28 completed gestational weeks until July 2008 and thereafter after 22 completed weeks. | *MBR* |
| Induction of labor | - Variable in *MBR*: Induced cesarean section - ICD-10: O61 - ICD-9: 659A, 659B | *MBR* |
| Cesarean section (any) | Variable in *MBR:* Cesarean section | *MBR* |
| Elective cesarean section | Variable in *MBR:* Planned cesarean section | *MBR* |
| Emergency cesarean section | Variables in *MBR:*   - Induced cesarean section - Spontaneous cesarean section | *MBR* |
| Instrumental delivery | Variables in *MBR* | *MBR* |

Abbreviations: *MBR*, *Medical Birth Register*; ICD, International Classification of Diseases; *NPR*, *National Patient Register*.

**Table S4:** Covariates included in the multivariable models.

| Covariate | Categories | Data source | Codes/definitions |
| --- | --- | --- | --- |
| Maternal age at delivery [years] | <25  25-35  ≥35 | *MBR* | - |
| Calendar year of delivery | 1990-1999  2000-2010  2011-2017 | *MBR* | - |
| Parity | Nulliparous (0 pregnancies prior to current pregnancy)  Multiparous (≥1 pregnancies prior to current pregnancy) | *MBR* | - |
| Level of education [years] | ≤9  10-12  ≥13 years  Missing | *LISA* | - |
| Country of birth | Nordic  Other  Missing | *Total Population Register* | - |
| BMI in early pregnancy [kg/m^2^] | <18.5  18.5 to <25  25 to <30  ≥30 | *MBR* | - |
| Smoking in early pregnancy | yes/no | *MBR* | - |
| Any diabetes (pre-existing type 1 or 2 diabetes or gestational diabetes) | yes/no | *MBR* | Type 1 or 2 diabetes (within 5 years prior to delivery):  ICD-10: E10, E11  ICD-9: 250  ICD-8: 250  Gestational diabetes (any time prior to delivery):  ICD-10: O24.4  ICD-9: 648W |
| Any hypertension (pre-existing hypertension or gestational hypertension) | yes/no | *MBR* | Hypertension (within 5 years prior to delivery):  ICD-10: I10-I16, I13-P  ICD-9: 401, 402, 403, 404, 405  ICD-8: 400-404  Gestational hypertension (any time prior to delivery):  ICD-10: O13  ICD-9: 760A |
| Dyslipidemia | yes/no | *NPR* and *PDR* | Within 5 years prior to delivery:  ICD-10: E78  ICD-9: 272  ICD-8: 272  ATC: C10AA, C10BA, C10BX, C10AB, C10AC |
| Pre-eclampsia | yes/no | *MBR* and *NPR* | Any time prior to delivery:  ICD-10: O14-O15  ICD-9: 642E, 642F, 642G, 642H |

Abbreviations: *MBR*, *Medical Birth Register*; *LISA*, Swedish Longitudinal Integrated Database for Health Insurance and Labour Market Studies (*Longitudinell Integrationsdatabas för Sjukförsäkrings- och Arbetsmarknadsstudier*); BMI, body mass index; ICD, International Classification of Diseases; *NPR*, *National Patient Register*; *PDR*, *Prescribed Drug Register*; ATC, Anatomical Therapeutic Chemical.

**Table S5:** Baseline characteristics of A. births in women with simple steatosis only vs. matched births in reference women without known MASLD, and baseline characteristics of B. births in women with simple steatosis plus MASH without fibrosis, noncirrhotic fibrosis, or cirrhosis vs. matched births in reference women without known MASLD.

|  | **A.**  **Simple steatosis** | | **B.**  **MASH / fibrosis / cirrhosis** | |
| --- | --- | --- | --- | --- |
|  | **Births in women with simple steatosis**  (n births = 175) | **Births in reference women**  (n births = 836) | **Births in women with MASH / fibrosis / cirrhosis**  (n births = 65) | **Births in reference women**  (n births = 304) |
| **Women, n** | 117 | 834 | 45 | 304 |
| **Maternal age at delivery [years]** |  |  |  |  |
| Mean (SD) | 31.9 (5.2) | 31.9 (5.2) | 30.5 (6.4) | 30.3 (6.2) |
| Median (IQR) | 32 (28-36) | 32 (28-36) | 30 (27-35) | 30 (27-35) |
| Range, min-max | 19-43 | 19-43 | 18-43 | 18-43 |
| 15 to <25 | 13 (7) | 61 (7) | 13 (20) | 62 (20) |
| 25 to <35 | 102 (58) | 485 (58) | 33 (51) | 158 (52) |
| 35 to 44 | 60 (34) | 290 (35) | 19 (29) | 84 (28) |
| **Pregnancy duration [days]** |  |  |  |  |
| Mean (SD) | 271.5 (17.6) | 278.5 (13.0) | 270.6 (19.6) | 279.2 (16.1) |
| Median (IQR) | 274 (264-283) | 280 (273-286) | 275 (264-283) | 282 (275-288) |
| Range, min-max | 188-305 | 177-300 | 189-297 | 163-301 |
| **Calendar year of delivery** |  |  |  |  |
| 1992-1999 | 55 (31) | 266 (32) | 10 (15) | 48 (16) |
| 2000-2010 | 89 (51) | 426 (51) | 36 (55) | 169 (56) |
| 2011-2017 | 31 (18) | 144 (17) | 19 (29) | 87 (29) |
| **Year of first MASLD diagnosis (index liver biopsy)** |  |  |  |  |
| Before 1999 | 128 (73) | - | 34 (52) | - |
| 2000-2010 | 43 (25) | - | 28 (43) | - |
| 2011-2017 | 4 (2) | - | 3 (5) | - |
| **Disease duration (time between first MASLD diagnosis and delivery) [years]** |  |  |  |  |
| Mean (SD) | 7.0 (4.9) | - | 7.0 (5.5) | - |
| Median (IQR) | 5.8 (3.1-9.9) | - | 5.2 (3.2-9.9) | - |
| Range, min-max | 0.0-26.5 | - | 0.7-25.2 | - |
| <5 | 75 (43) | - | 27 (42) | - |
| 5-10 | 58 (33) | - | 23 (35) | - |
| ≥10 | 42 (24) | - | 15 (23) | - |
| **Maternal country of birth** |  |  |  |  |
| Nordic | 153 (87) | 699 (84) | 50 (77) | 246 (81) |
| Other | 22 (13) | 137 (16) | 15 (23) | 58 (19) |
| **Civil status of the mother** |  |  |  |  |
| Living with partner | 150 (86) | 757 (91) | 57 (88) | 270 (89) |
| Not living with partner | 9 (5) | 14 (2) | 3 (5) | 7 (2) |
| Missing | 16 (9) | 65 (8) | 5 (8) | 27 (9) |
| **Level of education** |  |  |  |  |
| Compulsory school (≤9 years) | 26 (15) | 83 (10) | 10 (15) | 34 (11) |
| Upper secondary school (10-12 years) | 106 (61) | 370 (44) | 34 (52) | 119 (39) |
| College or university (≥13 years) | 43 (25) | 368 (44) | 21 (32) | 144 (47) |
| Missing | 0 (0) | 15 (2) | 0 (0) | 7 (2) |
| **Parity** |  |  |  |  |
| Nulliparous | 62 (35) | 296 (35) | 31 (48) | 146 (48) |
| Multiparous | 113 (65) | 540 (65) | 34 (52) | 158 (52) |
| **BMI in early pregnancy [kg/m^2^]** |  |  |  |  |
| Mean (SD) | 29.5 (6.0) | 24.6 (4.4) | 28.8 (5.4) | 24.9 (5.2) |
| Median (IQR) | 28.9 (25.0-33.3) | 24.0 (21.4-26.8) | 28.0 (25.0-32.4) | 23.7 (21.6-27.1) |
| Range, min-max | 18.9-48.2 | 17.4-43.0 | 19.4-42.4 | 17.3-61.3 |
| <18.5 | 0 (0) | 18 (2) | 0 (0) | 6 (2) |
| 18.5 to <25 | 40 (23) | 431 (52) | 15 (23) | 160 (53) |
| 25 to <30 | 49 (28) | 203 (24) | 23 (35) | 69 (23) |
| ≥30 | 70 (40) | 84 (10) | 22 (34) | 33 (11) |
| Missing | 16 (9) | 100 (12) | 5 (8) | 36 (12) |
| **Smoking in early pregnancy** |  |  |  |  |
| No | 135 (77) | 698 (83) | 52 (80) | 269 (88) |
| Yes | 33 (19) | 94 (11) | 8 (12) | 21 (7) |
| Missing | 7 (4) | 44 (5) | 5 (8) | 14 (5) |
| **Prior comorbidities and conditions** |  |  |  |  |
| Diabetes (including gestational diabetes) | 12 (6.9) | 9 (1.1) | 13 (20.0) | 1 (0.3) |
| Hypertension (including gestational hypertension) | 9 (5.1) | 4 (0.5) | 3 (4.6) | 2 (0.7) |
| Dyslipidemia | 3 (1.7) | 1 (0.1) | 1 (1.5) | 1 (0.3) |
| Pre-eclampsia | 9 (5.1) | 22 (2.6) | 6 (9.2) | 9 (3.0) |

Note: Data are presented as number (%) except where indicated otherwise.

Abbreviations: MASLD, metabolic dysfunction-associated steatotic liver disease; MASH, metabolic dysfunction-associated steatohepatitis; n, number; SD, standard deviation; IQR, interquartile range; min, minimum, max, maximum; BMI, body mass index.

*Description of baseline characteristics: Compared to simple steatosis, those with more severe MASLD were younger at delivery (30 vs. 32 years), were more frequently born in a non-Nordic country (23% vs. 13%), were more often nulliparous (48% vs. 35%), had higher rates of prior diabetes (20.0% vs. 6.9%) and pre-eclampsia (9.2% vs. 5.1%), but had similar rates of prior hypertension (4.6% vs. 5.1%), prior dyslipidemia (1.5% vs. 1.7%), and overweight or obesity (BMI ≥25 kg/m^2^) in early pregnancy (69% vs. 68%).

**Table S6:** Pregnancy and birth outcomes for all births in women with simple steatosis only versus births in reference women.

|  | **Births in women with simple steatosis**  n (%) | **Births in reference women**  n (%) | **OR* (95% CI)** | **OR** (95% CI)** |
| --- | --- | --- | --- | --- |
| **Live births, n** | 175 | 833 |  |  |
| **Preterm birth** |  |  |  |  |
| **Any preterm birth (<37 weeks)** | 31 (17.7%) | 38 (4.6%) | 4.55 (2.71-7.64) | 4.30 (2.32-7.96) |
| **Medically indicated** | 16 (9.1%) | 12 (1.4%) | 6.36 (3.01-13.45) | NE |
| **Spontaneous** | 15 (8.6%) | 25 (3.0%) | NE | NE |
| **Very preterm birth (<32 weeks)** | 5 (2.9%) | 6 (0.7%) | 4.28 (1.23-14.95) | NE |
| **Birth weight parameters** |  |  |  |  |
| **Small for gestational age (SGA)** | 24 (13.7%) | 66 (7.9%) | 1.89 (1.14-3.14) | 1.60 (0.90-2.84) |
| **Large for gestational age (LGA)** | 32 (18.3%) | 103 (12.4%) | 1.59 (1.02-2.47) | 1.06 (0.66-1.70) |
| **Low birth weight (<2,500 g)** |  |  |  |  |
| **All live births** | 20 (11.4%) | 25 (3.0%) | 4.90 (2.51-9.60) | NE |
| **Term births** | 3 (2.1%) | 11 (1.4%) | 2.35 (0.59-9.45) | NE |
| **Missing information** | 1 (0.6%) | 3 (0.4%) |  |  |
| **All births, n** | 175 | 836 |  |  |
| **Pregnancy and maternal outcomes** |  |  |  |  |
| **Induction of labor** | 33 (18.9%) | 119 (14.2%) | 1.42 (0.92-2.18) | 1.13 (0.72-1.78) |
| **Cesarean section** | 56 (32.0%) | 130 (15.6%) | 2.73 (1.85-4.03) | 1.72 (1.18-2.52) |
| **Elective** | 16 (9.1%) | 55 (6.6%) | 1.47 (0.81-2.65) | 0.94 (0.48-1.84) |
| **Emergency** | 35 (20.0%) | 71 (8.5%) | 2.97 (1.87-4.73) | 2.17 (1.30-3.65) |

Abbreviations: n, number; OR, odds ratio; NE, not estimable.

*Model 1: Conditioned on the matching set (maternal age at delivery, calendar year of delivery, and parity).

**Model 2: Conditioned on the matching set and additionally adjusted for level of education, country of birth, BMI in early pregnancy, smoking in early pregnancy, and the following conditions prior to delivery: diabetes (type 1, type 2, and gestational diabetes), hypertension (including gestational hypertension), dyslipidemia, and pre-eclampsia.

**Table S7:** Pregnancy and birth outcomes for all births in women with MASH without fibrosis, noncirrhotic fibrosis, or cirrhosis versus births in reference women.

|  | **Births in women with MASH / fibrosis / cirrhosis**  n (%) | **Births in reference women**  n (%) | **OR* (95% CI)** | **OR** (95% CI)** |
| --- | --- | --- | --- | --- |
| **Live births, n** | 64 | 303 |  |  |
| **Preterm birth** |  |  |  |  |
| **Any preterm birth (<37 weeks)** | 9 (14.1%) | 15 (5.0%) | 3.59 (1.44-8.96) | 1.53 (0.23-10.02) |
| **Medically indicated** | 7 (10.9%) | 2 (0.7%) | 17.09 (3.55-82.34) | NE |
| **Spontaneous** | 2 (3.1%) | 13 (4.3%) | NE | NE |
| **Very preterm birth (<32 weeks)** | 2 (3.1%) | 3 (1.0%) | 3.33 (0.56-19.95) | NE |
| **Birth weight parameters** |  |  |  |  |
| **Small for gestational age (SGA)** | 11 (17.2%) | 31 (10.2%) | 1.72 (0.82-3.63) | 1.73 (0.66-4.55) |
| **Large for gestational age (LGA)** | 16 (25.0%) | 33 (10.9%) | 2.67 (1.35-5.30) | 1.43 (0.58-3.53) |
| **Low birth weight (<2,500 g)** |  |  |  |  |
| **All live births** | 6 (9.4%) | 13 (4.3%) | 2.35 (0.84-6.60) | NE |
| **Term births** | 2 (3.6%) | 5 (1.7%) | 2.91 (0.49-17.47) | NE |
| **Missing information** | 0 (0.0%) | 1 (0.3%) |  |  |
| **All births, n** | 65 | 304 |  |  |
| **Pregnancy and maternal outcomes** |  |  |  |  |
| **Induction of labor** | 17 (26.2%) | 40 (13.2%) | 2.25 (1.18-4.28) | 1.83 (0.85-3.92) |
| **Cesarean section** | 21 (32.3%) | 52 (17.1%) | 2.63 (1.36-5.09) | 1.52 (0.70-3.28) |
| **Elective** | 11 (16.9%) | 28 (9.2%) | 2.24 (0.99-5.09) | 1.93 (0.71-5.23) |
| **Emergency** | 10 (15.4%) | 24 (7.9%) | 2.25 (0.97-5.21) | 0.95 (0.27-3.40) |

Abbreviations: MASH, metabolic dysfunction-associated steatohepatitis; n, number; OR, odds ratio; CI, confidence interval; NE, not estimable.

*Model 1: Conditioned on the matching set (maternal age at delivery, calendar year of delivery, and parity).

**Model 2: Conditioned on the matching set and additionally adjusted for level of education, country of birth, BMI in early pregnancy, smoking in early pregnancy, and the following conditions prior to delivery: diabetes (type 1, type 2, and gestational diabetes), hypertension (including gestational hypertension), dyslipidemia, and pre-eclampsia.

**Table S8:** Baseline characteristics of women with MASLD and their female siblings giving birth*.

|  | **Births in women with MASLD**  (n births = 78) | **Births in siblings**  (n births = 125) |
| --- | --- | --- |
| **Women, n** | 45 | 59 |
| **Maternal age at delivery [years]** |  |  |
| Mean (SD) | 30.9 (5.2) | 28.6 (5.3) |
| Median (IQR) | 30 (27-35) | 28 (25-32) |
| Range, min-max | 19-43 | 19-42 |
| 15 to <25 | 8 (10) | 27 (22) |
| 25 to <35 | 48 (62) | 79 (63) |
| 35 to 44 | 22 (28) | 19 (15) |
| **Pregnancy duration [days]** |  |  |
| Mean (SD) | 270.0 (19.1) | 278.6 (11.5) |
| Median (IQR) | 272 (260-283) | 279 (272-287) |
| Range, min-max | 188-305 | 230-301 |
| **Calendar year of delivery** |  |  |
| 1992-1999 | 16 (21) | 43 (34) |
| 2000-2010 | 41 (53) | 67 (54) |
| 2011-2017 | 21 (27) | 15 (12) |
| **Year of MASLD diagnosis (index liver biopsy)** |  |  |
| Before 1999 | 49 (63) | - |
| 2000-2010 | 27 (35) | - |
| 2011-2017 | 2 (3) | - |
| **Disease duration (time between first MASLD diagnosis and delivery) [years]** |  |  |
| Mean (SD) | 7.0 (5.1) |  |
| Median (IQR) | 5.7 (3.0-9.2) |  |
| Range, min-max | 0.0-25.2 |  |
| <5 | 31 (40) | - |
| 5-10 | 31 (40) | - |
| ≥10 | 16 (21) | - |
| **Maternal country of birth** |  |  |
| Nordic | 75 (96) | 125 (100) |
| Other | 3 (4) | 0 (0) |
| **Civil status of the mother** |  |  |
| Living with partner | 65 (83) | 116 (93) |
| Not living with partner | 5 (6) | 3 (2) |
| Missing | 8 (10) | 6 (5) |
| **Level of education** |  |  |
| Compulsory school (≤9 years) | 7 (9) | 22 (18) |
| Upper secondary school (10-12 years) | 48 (62) | 81 (65) |
| College or university (≥13 years) | 23 (29) | 22 (18) |
| Missing | 0 (0) | 0 (0) |
| **Parity** |  |  |
| Nulliparous | 30 (38) | 46 (37) |
| Multiparous | 48 (62) | 79 (63) |
| **BMI in early pregnancy [kg/m^2^]** |  |  |
| Mean (SD) | 30.1 (6.6) | 26.6 (6.0) |
| Median (IQR) | 29.3 (25.7-35.4) | 25.0 (22.2-28.7) |
| Range, min-max | 18.9-48.2 | 18.8-47.9 |
| <18.5 | 0 (0) | 0 (0) |
| 18.5 to <25 | 15 (19) | 55 (44) |
| 25 to <30 | 26 (33) | 33 (26) |
| ≥30 | 30 (38) | 23 (18) |
| Missing | 7 (9) | 14 (11) |
| **Smoking in early pregnancy** |  |  |
| No | 66 (85) | 106 (85) |
| Yes | 9 (12) | 13 (10) |
| Missing | 3 (4) | 6 (5) |
| **Prior comorbidities and conditions** |  |  |
| Diabetes (including gestational diabetes) | 8 (10.3) | 4 (3.2) |
| Hypertension (including gestational hypertension) | 2 (2.6) | 1 (0.8) |
| Dyslipidemia | 1 (1.3) | 0 (0.0) |
| Pre-eclampsia | 6 (7.7) | 4 (3.2) |

Note: Data are presented as number (%) except where indicated otherwise.

Abbreviations: MASLD, metabolic dysfunction-associated steatotic liver disease; n, number; SD, standard deviation; IQR, interquartile range; min, minimum, max, maximum; BMI, body mass index.

*Description of baseline characteristics: Women with MASLD in the sibling analyses, compared to the main analyses, were younger (30 vs. 32 years), were more often born in a Nordic country (96% vs. 85%), were better educated (≥10 years: 91% vs. 85%), smoked less in early pregnancy (12% vs. 17%), and had a lower rate of prior hypertension (2.6% vs. 5.0%) and dyslipidemia (1.3% vs. 1.7%), but more prior pre-eclampsia (7.7% vs. 6.2%).

**Table S9:** Pregnancy and birth outcomes for births in women with MASLD versus births by their siblings.

|  | **Births in women with MASLD**  n (%) | **Births in siblings**  n (%) | **OR* (95% CI)** | **OR** (95% CI)** |
| --- | --- | --- | --- | --- |
| **Live births, n** | 78 | 125 |  |  |
| **Preterm birth** |  |  |  |  |
| **Any preterm birth (<37 weeks)** | 16 (20.5%) | 4 (3.2%) | 14.85 (3.28-67.17) | 18.92 (3.83-93.54) |
| **Medically indicated** | 9 (11.5%) | 3 (2.4%) | 4.78 (1.16-19.65) | 6.34 (1.08-37.19) |
| **Spontaneous** | 7 (9.0%) | 1 (0.8%) | 542.58 (1.40-211029.21) | 486.12 (0.93-253899.08) |
| **Very preterm birth (<32 weeks)** | 2 (2.6%) | 0 (0.0%) | NE | NE |
| **Cesarean section** | 32 (41.0%) | 24 (19.2%) | 3.09 (1.42-6.70) | 1.94 (1.02-3.71) |
| **Elective** | 9 (11.5%) | 10 (8.0%) | 2.00 (0.65-6.08) | 2.46 (0.75-8.10) |
| **Emergency** | 21 (26.9%) | 14 (11.2%) | 3.61 (1.38-9.44) | 2.56 (1.04-6.25) |

Abbreviations: MASLD, metabolic dysfunction-associated steatotic liver disease; n, number; OR, odds ratio; CI, confidence interval; NE, not estimable.

*Model 1: Conditioned on the matching set (maternal age at delivery, calendar year of delivery, and parity).

**Model 2 (restricted): Conditioned on the matching set and additionally adjusted for BMI in early pregnancy, and the following conditions prior to delivery: diabetes (type 1, type 2, and gestational diabetes), hypertension (including gestational hypertension).

**Table S10:** Pregnancy and birth outcomes for births in women with MASLD versus births in reference women with a BMI <30 kg/m^2^ in early pregnancy.

|  | **Births in women with MASLD**  n (%) | **Births in reference women**  n (%) | **OR* (95% CI)** | **OR** (95% CI)** |
| --- | --- | --- | --- | --- |
| **Live births, n** | 239 | 883 |  |  |
| **Preterm birth** |  |  |  |  |
| **Any preterm birth (<37 weeks)** | 40 (16.7%) | 37 (4.2%) | 4.89 (2.95-8.12) | 3.03 (1.33-6.91) |
| **Medically indicated** | 23 (9.6%) | 9 (1.0%) | 9.86 (4.55-21.39) | NE |
| **Spontaneous** | 17 (7.1%) | 28 (3.2%) | 2.21 (1.17-4.17) | 2.88 (1.01-8.22) |
| **Very preterm birth (<32 weeks)** | 7 (2.9%) | 7 (0.8%) | 4.37 (1.44-13.25) | NE |
| **Cesarean section** | 77 (32.1%) | 136 (15.3%) | 3.02 (2.12-4.31) | 1.73 (1.08-2.79) |
| **Elective** | 27 (11.2%) | 63 (7.1%) | 1.82 (1.10-3.01) | 0.98 (0.39-2.46) |
| **Emergency** | 45 (18.8%) | 71 (8.0%) | 3.00 (1.96-4.59) | 2.27 (1.21-4.24) |

Abbreviations: MASLD, metabolic dysfunction-associated steatotic liver disease; BMI, body mass index; n, number; OR, odds ratio; CI, confidence interval; NE, not estimable.

*Model 1: Conditioned on the matching set (maternal age at delivery, calendar year of delivery, and parity).

**Model 2: Conditioned on the matching set and additionally adjusted for level of education, country of birth, BMI in early pregnancy, smoking in early pregnancy, and the following conditions prior to delivery: diabetes (type 1, type 2, and gestational diabetes), hypertension (including gestational hypertension), dyslipidemia, and pre-eclampsia.

**Table S11:** Pregnancy and birth outcomes for births in women with MASLD versus births in reference women with a BMI ≥25 kg/m^2^ in early pregnancy.

|  | **Births in women with MASLD**  n (%) | **Births in reference women**  n (%) | **OR* (95% CI)** | **OR** (95% CI)** |
| --- | --- | --- | --- | --- |
| **Live births, n** | 239 | 389 |  |  |
| **Preterm birth** |  |  |  |  |
| **Any preterm birth (<37 weeks)** | 40 (16.7%) | 16 (4.1%) | 6.45 (3.15-13.21) | 4.60 (2.00-10.60) |
| **Medically indicated** | 23 (9.6%) | 5 (1.3%) | 9.07 (3.38-24.35) | NE |
| **Spontaneous** | 17 (7.1%) | 11 (2.8%) | 3.10 (1.28-7.51) | 5.34 (1.26-22.71) |
| **Very preterm birth (<32 weeks)** | 7 (2.9%) | 2 (0.5%) | 7.53 (1.48-38.32) | NE |
| **Cesarean section** | 77 (32.1%) | 86 (22.1%) | 1.83 (1.21-2.76) | 1.20 (0.77-1.86) |
| **Elective** | 27 (11.2%) | 42 (10.8%) | 1.09 (0.62-1.92) | 1.03 (0.54-1.96) |
| **Emergency** | 45 (18.8%) | 43 (11.0%) | 2.30 (1.34-3.94) | 1.35 (0.67-2.75) |

Abbreviations: MASLD, metabolic dysfunction-associated steatotic liver disease; BMI, body mass index; n, number; OR, odds ratio; CI, confidence interval; NE, not estimable.

*Model 1: Conditioned on the matching set (maternal age at delivery, calendar year of delivery, and parity).

**Model 2: Conditioned on the matching set and additionally adjusted for level of education, country of birth, BMI in early pregnancy, smoking in early pregnancy, and the following conditions prior to delivery: diabetes (type 1, type 2, and gestational diabetes), hypertension (including gestational hypertension), dyslipidemia, and pre-eclampsia.

**Table S12:** Pregnancy and birth outcomes for births in nulliparous women with MASLD versus births in reference women.

|  | **Births in women with MASLD**  n (%) | **Births in reference women**  n (%) | **OR* (95% CI)** | **OR** (95% CI)** |
| --- | --- | --- | --- | --- |
| **Live births, n** | 92 | 441 |  |  |
| **Preterm birth** |  |  |  |  |
| **Any preterm birth (<37 weeks)** | 16 (17.4%) | 24 (5.4%) | 3.80 (1.90-7.60) | 3.19 (1.28-7.94) |
| **Medically indicated** | 8 (8.7%) | 6 (1.4%) | 6.36 (2.20-18.35) | NE |
| **Spontaneous** | 8 (8.7%) | 17 (3.9%) | 2.45 (1.03-5.80) | 2.54 (0.76-8.41) |
| **Very preterm birth (<32 weeks)** | 5 (5.4%) | 4 (0.9%) | 5.95 (1.60-22.19) | NE |
| **Birth weight parameters** |  |  |  |  |
| **Small for gestational age (SGA)** | 19 (20.7%) | 56 (12.7%) | 1.72 (0.97-3.06) | 1.40 (0.72-2.72) |
| **Large for gestational age (LGA)** | 17 (18.5%) | 35 (7.9%) | 2.57 (1.35-4.91) | 1.44 (0.64-3.24) |
| **Low birth weight (<2,500 g)** |  |  |  |  |
| **All live births** | 12 (13.0%) | 20 (4.5%) | 3.12 (1.46-6.67) | 1.45 (0.42-5.00) |
| **Term births** | 2 (2.6%) | 10 (2.4%) | 1.32 (0.27-6.37) | NE |
| **Missing information** | 0 (0.0%) | 1 (0.2%) |  |  |
| **Other neonatal outcomes** |  |  |  |  |
| **Apgar <7 at 5 minutes** | 2 (2.2%) | 8 (1.8%) | 1.14 (0.24-5.37) | 0.21 (0.03-1.73) |
| **Missing information** | 1 (1.1%) | 3 (0.7%) |  |  |
| **Congenital malformations** | 10 (10.9%) | 27 (6.1%) | 1.84 (0.86-3.98) | 1.64 (0.63-4.31) |
| **Missing information** | 1 (1.1%) | 1 (0.2%) |  |  |
| **Neonatal death** | 0 (0.0%) | 0 (0.0%) | NA (NA-NA) | NE |
| **All births, n** | 93 | 442 |  |  |
| **Intrauterine fetal death** |  |  |  |  |
| **Stillbirth** | 1 (1.1%) | 1 (0.2%) | 5.00 (0.31-79.94) | NE |
| **Pregnancy and maternal outcomes** |  |  |  |  |
| **Induction of labor** | 19 (20.4%) | 73 (16.5%) | 1.31 (0.74-2.31) | 1.12 (0.62-2.04) |
| **Cesarean section** | 36 (38.7%) | 88 (19.9%) | 2.70 (1.63-4.47) | 1.77 (1.06-2.97) |
| **Elective** | 17 (18.3%) | 56 (12.7%) | 1.62 (0.88-3.00) | 1.23 (0.61-2.48) |
| **Emergency** | 17 (18.3%) | 30 (6.8%) | 3.40 (1.74-6.63) | 2.67 (0.98-7.32) |
| **Instrumental delivery** | 11 (11.8%) | 66 (14.9%) | 0.75 (0.38-1.49) | 0.82 (0.39-1.70) |

Abbreviations: MASLD, metabolic dysfunction-associated steatotic liver disease; n, number; OR, odds ratio; CI, confidence interval; NE, not estimable.

*Model 1: Conditioned on the matching set (maternal age at delivery, calendar year of delivery, and parity).

**Model 2: Conditioned on the matching set and additionally adjusted for level of education, country of birth, BMI in early pregnancy, smoking in early pregnancy, and the following conditions prior to delivery: diabetes (type 1, type 2, and gestational diabetes), hypertension (including gestational hypertension), dyslipidemia, and pre-eclampsia.

**Table S13:** Pregnancy and birth outcomes for births in parous women with MASLD versus births in reference women.

|  | **Births in women with MASLD**  n (%) | **Births in reference women**  n (%) | **OR* (95% CI)** | **OR** (95% CI)** |
| --- | --- | --- | --- | --- |
| **Live births, n** | 147 | 695 |  |  |
| **Preterm birth** |  |  |  |  |
| **Any preterm birth (<37 weeks)** | 24 (16.3%) | 29 (4.2%) | 4.70 (2.59-8.51) | 4.63 (2.16-9.91) |
| **Medically indicated** | 15 (10.2%) | 8 (1.2%) | 9.03 (3.83-21.31) | NE |
| **Spontaneous** | 9 (6.1%) | 21 (3.0%) | 2.16 (0.95-4.89) | 2.84 (0.95-8.46) |
| **Very preterm birth (<32 weeks)** | 2 (1.4%) | 5 (0.7%) | 2.04 (0.36-11.39) | NE |
| **Cesarean section** | 41 (27.9%) | 94 (13.5%) | 2.71 (1.72-4.25) | 1.79 (1.13-2.82) |
| **Elective** | 10 (6.8%) | 27 (3.9%) | 1.79 (0.85-3.78) | 1.07 (0.42-2.68) |
| **Emergency** | 28 (19.0%) | 65 (9.3%) | 2.48 (1.48-4.13) | 1.96 (1.13-3.41) |

Abbreviations: MASLD, metabolic dysfunction-associated steatotic liver disease; n, number; OR, odds ratio; CI, confidence interval; NE, not estimable.

*Model 1: Conditioned on the matching set (maternal age at delivery, calendar year of delivery, and parity).

**Model 2: Conditioned on the matching set and additionally adjusted for level of education, country of birth, BMI in early pregnancy, smoking in early pregnancy, and the following conditions prior to delivery: diabetes (type 1, type 2, and gestational diabetes), hypertension (including gestational hypertension), dyslipidemia, and pre-eclampsia.

**Table S14:** Any preterm birth and cesarean section for births in women with MASLD versus births in reference women by maternal disease duration before delivery.

|  | **Births in women with MASLD**  n (%) | **Births in reference women**  n (%) | **OR* (95% CI)** | **OR** (95% CI)** |
| --- | --- | --- | --- | --- |
| **Live births, n** | 147 | 695 |  |  |
| **Any preterm birth (<37 weeks)** | 53 (4.7%) | 40 (16.7%) | 4.30 (2.74-6.74) | 3.38 (2.08-5.48) |
| Maternal disease duration <5 years | 22 (5.0%) | 18 (19.8%) | 4.75 (2.37-9.51) | 4.13 (1.77-9.63) |
| Maternal disease duration ≥5 years | 25 (4.6%) | 19 (16.8%) | 4.41 (2.29-8.49) | 6.30 (2.31-17.22) |
| **All births, n** | 1140 | 240 |  |  |
| **Cesarean section** | 182 (16.0%) | 77 (32.1%) | 2.70 (1.93-3.78) | 1.78 (1.30-2.42) |
| Maternal disease duration <5 years | 62 (14.4%) | 26 (28.9%) | 2.67 (1.50-4.77) | 1.82 (1.02-3.25) |
| Maternal disease duration ≥5 years | 89 (16.1%) | 40 (35.1%) | 3.29 (2.00-5.42) | 2.32 (1.46-3.69) |

Abbreviations: MASLD, metabolic dysfunction-associated steatotic liver disease; n, number; OR, odds ratio; CI, confidence interval.

*Model 1: Conditioned on the matching set (maternal age at delivery, calendar year of delivery, and parity).

**Model 2: Conditioned on the matching set and additionally adjusted for level of education, country of birth, BMI in early pregnancy, smoking in early pregnancy, and the following conditions prior to delivery: diabetes (type 1, type 2, and gestational diabetes), hypertension (including gestational hypertension), dyslipidemia, and pre-eclampsia.

**Table S15:** Pregnancy and birth outcomes for births in women with MASLD versus births in reference women without known MASLD in the main analysis (model 1 and 2) and in *post-hoc* sensitivity analyses (model 3 and 4) which consider different time periods of covariate measurement than in the main analysis.

|  |  |  | **Main analysis** | | **Metabolic-related covariates measured any time prior to index liver biopsy** | **Metabolic-related covariates measured any time prior to start of pregnancy** |
| --- | --- | --- | --- | --- | --- | --- |
|  | **Births in women with MASLD**  n (%) | **Births in reference women**  n (%) | **OR (95% CI)**  Model 1* | **OR (95% CI)**  Model 2** | **OR (95% CI)**  Model 3*** | **OR (95% CI)**  Model 4**** |
| **Live births, n** | 239 | 1136 |  |  |  |  |
| **Preterm birth** |  |  |  |  |  |  |
| **Any preterm birth (<37 weeks)** | 40 (16.7%) | 53 (4.7%) | 4.30 (2.74-6.74) | 3.41 (1.98-5.88) | 4.11 (2.39-7.05) | 4.00 (2.31-6.93) |
| **Medically indicated** | 23 (9.6%) | 14 (1.2%) | 7.88 (4.05-15.33) | 11.90 (2.46-57.59) | 22.23 (5.27-93.76) | 27.35 (5.94-125.95) |
| **Spontaneous** | 17 (7.1%) | 38 (3.3%) | 2.29 (1.26-4.15) | 2.42 (1.16-5.04) | 2.39 (1.16-4.93) | 2.14 (1.03-4.48) |
| **Very preterm birth (<32 weeks)** | 7 (2.9%) | 9 (0.8%) | 3.94 (1.42-10.94) | 1.74 (0.22-13.65) | 4.38 (0.90-21.27) | 5.11 (0.90-29.07) |
| **Pre-eclampsia** | 15 (6.2%) | 31 (2.7%) | 2.29 (1.23-4.30) | 1.48 (0.65-3.37) | 1.57 (0.74-3.34) | 1.62 (0.71-3.68) |
| **Birth weight parameters** |  |  |  |  |  |  |
| **Small for gestational age (SGA)** | 35 (14.6%) | 97 (8.5%) | 1.84 (1.21-2.79) | 1.52 (0.94-2.43) | 1.60 (1.02-2.52) | 1.65 (1.04-2.60) |
| **Large for gestational age (LGA)** | 48 (20.1%) | 136 (12.0%) | 1.84 (1.27-2.67) | 1.17 (0.78-1.74) | 1.13 (0.76-1.69) | 1.16 (0.78-1.72) |
| **Low birth weight (<2,500 g)** |  |  |  |  |  |  |
| **All live births** | 26 (10.9%) | 38 (3.3%) | 3.92 (2.25-6.83) | 2.66 (1.35-5.24) | 3.81 (1.97-7.38) | 4.06 (2.10-7.85) |
| **Term births** | 5 (2.5%) | 16 (1.5%) | 2.55 (0.85-7.62) | 1.25 (0.28-5.48) | 1.04 (0.23-4.65) | 1.01 (0.19-5.40) |
| **Missing information** | 1 (0.4%) | 4 (0.4%) |  |  |  |  |
| **Macrosomia (>4,000 g)** |  |  |  |  |  |  |
| **All live births** | 46 (19.2%) | 233 (20.5%) | 0.90 (0.62-1.29) | 0.72 (0.50-1.04) | 0.72 (0.50-1.04) | 0.73 (0.51-1.05) |
| **Term births** | 44 (22.1%) | 233 (21.5%) | 1.01 (0.69-1.49) | 0.79 (0.55-1.15) | 0.81 (0.56-1.18) | 0.83 (0.58-1.20) |
| **Other neonatal outcomes** |  |  |  |  |  |  |
| **Apgar <7 at 5 minutes** | 4 (1.7%) | 15 (1.3%) | 1.25 (0.42-3.78) | 1.74 (0.39-7.82) | 1.57 (0.39-6.28) | 1.29 (0.35-4.78) |
| **Missing information** | 2 (0.8%) | 9 (0.8%) |  |  |  |  |
| **Congenital malformations** | 16 (6.7%) | 61 (5.4%) | 1.26 (0.71-2.26) | 1.06 (0.57-2.00) | 1.14 (0.61-2.14) | 1.07 (0.56-2.04) |
| **Missing information** | 1 (0.4%) | 4 (0.4%) |  |  |  |  |
| **Neonatal death** | 1 (0.4%) | 2 (0.2%) | 2.50 (0.23-27.57) | NE | NE | NE |
| **All births, n** | 240 | 1140 |  |  |  |  |
| **Intrauterine fetal death** |  |  |  |  |  |  |
| **Stillbirth** | 1 (0.4%) | 4 (0.4%) | 1.11 (0.12-10.06) | NE | NE | NE |
| **Pregnancy and maternal outcomes** |  |  |  |  |  |  |
| **Induction of labor** | 50 (20.8%) | 159 (13.9%) | 1.63 (1.14-2.32) | 1.27 (0.87-1.85) | 1.40 (0.97-2.01) | 1.36 (0.94-1.96) |
| **Cesarean section** | 77 (32.1%) | 182 (16.0%) | 2.70 (1.93-3.78) | 1.63 (1.17-2.27) | 1.74 (1.26-2.41) | 1.64 (1.18-2.27) |
| **Elective** | 27 (11.2%) | 83 (7.3%) | 1.69 (1.05-2.72) | 1.18 (0.70-2.00) | 1.18 (0.71-1.96) | 1.13 (0.66-1.91) |
| **Emergency** | 45 (18.8%) | 95 (8.3%) | 2.78 (1.85-4.18) | 1.90 (1.20-3.02) | 2.14 (1.37-3.35) | 2.05 (1.31-3.22) |
| **Instrumental delivery** | 15 (6.2%) | 83 (7.3%) | 0.83 (0.46-1.50) | 0.84 (0.45-1.59) | 0.85 (0.45-1.61) | 0.84 (0.44-1.58) |

Abbreviations: MASLD, metabolic dysfunction-associated steatotic liver disease; n, number; OR, odds ratio; CI, confidence interval; NE, not estimable.

*Model 1: Conditioned on the matching set (maternal age at delivery, calendar year of delivery, and parity).

**Model 2: Conditioned on the matching set and additionally adjusted for level of education, country of birth, BMI in early pregnancy, smoking in early pregnancy, and the following conditions prior to delivery: diabetes (type 1, type 2, and gestational diabetes), hypertension (including gestational hypertension), dyslipidemia, and pre-eclampsia.

***Model 3: Same as model 2, but covariate measurement of metabolic disorders any time prior to index liver biopsy (i.e., first biopsy indicating MASLD).

****Model 4: Same as model 2, but covariate measurement of metabolic disorders any time prior to the start of pregnancy of interest.

**Table S16:** Pregnancy and birth outcomes for births in women with MASLD versus births in reference women without known MASLD in the main analysis and after restricting to women without gestational diabetes during the pregnancy of interest (*post-hoc* sensitivity analysis).

|  | **Main analysis** | | | **Sensitivity analysis:**  **Births of women without gestational diabetes** | | |
| --- | --- | --- | --- | --- | --- | --- |
|  | **Births in women with MASLD**  n (%) | **Births in reference women**  n (%) | **OR (95% CI)**  Model 2** | **Births in women with MASLD**  n (%) | **Births in reference women**  n (%) | **OR (95% CI)**  Model 2** |
| **Live births, n** | 239 | 1136 |  | 217 | 1127 |  |
| **Preterm birth** |  |  |  |  |  |  |
| **Any preterm birth (<37 weeks)** | 40 (16.7%) | 53 (4.7%) | 3.41 (1.98-5.88) | 34 (15.7%) | 50 (4.4%) | 3.92 (2.13-7.21) |
| **Medically indicated** | 23 (9.6%) | 14 (1.2%) | 11.90 (2.46-57.59) | 20 (9.2%) | 12 (1.1%) | 108.62 (4.11-2872.24) |
| **Spontaneous** | 17 (7.1%) | 38 (3.3%) | 2.42 (1.16-5.04) | 14 (6.5%) | 37 (3.3%) | 2.31 (1.02-5.23) |
| **Very preterm birth (<32 weeks)** | 7 (2.9%) | 9 (0.8%) | 1.74 (0.22-13.65) | 6 (2.8%) | 9 (0.8%) | 1.38 (0.15-13.00) |
| **Pre-eclampsia** | 15 (6.2%) | 31 (2.7%) | 1.48 (0.65-3.37) | 13 (6.0%) | 30 (2.7%) | 1.35 (0.53-3.47) |
| **Birth weight parameters** |  |  |  |  |  |  |
| **Small for gestational age (SGA)** | 35 (14.6%) | 97 (8.5%) | 1.52 (0.94-2.43) | 34 (15.7%) | 94 (8.3%) | 1.65 (1.01-2.68) |
| **Large for gestational age (LGA)** | 48 (20.1%) | 136 (12.0%) | 1.17 (0.78-1.74) | 44 (20.3%) | 133 (11.8%) | 1.27 (0.84-1.94) |
| **Low birth weight (<2,500 g)** |  |  |  |  |  |  |
| **All live births** | 26 (10.9%) | 38 (3.3%) | 2.66 (1.35-5.24) | 23 (10.6%) | 36 (3.2%) | 2.68 (1.25-5.77) |
| **Term births** | 5 (2.5%) | 16 (1.5%) | 1.25 (0.28-5.48) | 5 (2.7%) | 16 (1.5%) | 1.58 (0.33-7.51) |
| **Missing information** | 1 (0.4%) | 4 (0.4%) |  | 1 (0.4%) | 4 (0.4%) |  |
| **Macrosomia (>4,000 g)** |  |  |  |  |  |  |
| **All live births** | 46 (19.2%) | 233 (20.5%) | 0.72 (0.50-1.04) | 43 (19.8%) | 230 (20.4%) | 0.78 (0.54-1.14) |
| **Term births** | 44 (22.1%) | 233 (21.5%) | 0.79 (0.55-1.15) | 42 (23.0%) | 230 (21.4%) | 0.89 (0.61-1.31) |
| **Other neonatal outcomes** |  |  |  |  |  |  |
| **Apgar <7 at 5 minutes** | 4 (1.7%) | 15 (1.3%) | 1.74 (0.39-7.82) | 4 (1.8%) | 15 (1.3%) | 1.74 (0.39-7.82) |
| **Missing information** | 2 (0.8%) | 9 (0.8%) |  | 2 (0.8%) | 9 (0.8%) |  |
| **Congenital malformations** | 16 (6.7%) | 61 (5.4%) | 1.06 (0.57-2.00) | 14 (6.5%) | 60 (5.3%) | 1.09 (0.57-2.11) |
| **Missing information** | 1 (0.4%) | 4 (0.4%) |  | 1 (0.4%) | 4 (0.4%) |  |
| **Neonatal death** | 1 (0.4%) | 2 (0.2%) | NE | 1 (0.5%) | 2 (0.2%) | NE |
| **All births, n** | 240 | 1140 |  | 218 | 1131 |  |
| **Intrauterine fetal death** |  |  |  |  |  |  |
| **Stillbirth** | 1 (0.4%) | 4 (0.4%) | NE | 1 (0.5%) | 4 (0.4%) | NE |
| **Pregnancy and maternal outcomes** |  |  |  |  |  |  |
| **Induction of labor** | 50 (20.8%) | 159 (13.9%) | 1.27 (0.87-1.85) | 42 (19.3%) | 157 (13.9%) | 1.25 (0.84-1.85) |
| **Cesarean section** | 77 (32.1%) | 182 (16.0%) | 1.63 (1.17-2.27) | 73 (33.5%) | 176 (15.6%) | 1.79 (1.27-2.52) |
| **Elective** | 27 (11.2%) | 83 (7.3%) | 1.18 (0.70-2.00) | 26 (11.9%) | 80 (7.1%) | 1.24 (0.72-2.15) |
| **Emergency** | 45 (18.8%) | 95 (8.3%) | 1.90 (1.20-3.02) | 42 (19.3%) | 92 (8.1%) | 2.21 (1.37-3.56) |
| **Instrumental delivery** | 15 (6.2%) | 83 (7.3%) | 0.84 (0.45-1.59) | 12 (5.5%) | 83 (7.3%) | 0.72 (0.36-1.43) |

Abbreviations: MASLD, metabolic dysfunction-associated steatotic liver disease; n, number; OR, odds ratio; CI, confidence interval; NE, not estimable.

*Model 1: Conditioned on the matching set (maternal age at delivery, calendar year of delivery, and parity).

**Model 2: Conditioned on the matching set and additionally adjusted for level of education, country of birth, BMI in early pregnancy, smoking in early pregnancy, and the following conditions prior to delivery: diabetes (type 1, type 2, and gestational diabetes), hypertension (including gestational hypertension), dyslipidemia, and pre-eclampsia. In sensitivity analysis (restricted to births of women without gestational diabetes), only gestational diabetes prior to pregnancy of interested was included as covariate.

**Table S17:** Pregnancy and birth outcomes for births in women with MASLD versus births in reference women without known MASLD in the main analysis and after restricting to women without gestational hypertension during the pregnancy of interest (*post-hoc* sensitivity analysis).

|  | **Main analysis** | | | **Sensitivity analysis:**  **Births of women without gestational hypertension** | | |
| --- | --- | --- | --- | --- | --- | --- |
|  | **Births in women with MASLD**  n (%) | **Births in reference women**  n (%) | **OR (95% CI)**  Model 2** | **Births in women with MASLD**  n (%) | **Births in reference women**  n (%) | **OR (95% CI)**  Model 2** |
| **Live births, n** | 239 | 1136 |  | 234 | 1127 |  |
| **Preterm birth** |  |  |  |  |  |  |
| **Any preterm birth (<37 weeks)** | 40 (16.7%) | 53 (4.7%) | 3.41 (1.98-5.88) | 39 (16.7%) | 52 (4.6%) | 3.82 (2.15-6.77) |
| **Medically indicated** | 23 (9.6%) | 14 (1.2%) | 11.90 (2.46-57.59) | 22 (9.4%) | 13 (1.2%) | 31.39 (3.07-321.07) |
| **Spontaneous** | 17 (7.1%) | 38 (3.3%) | 2.42 (1.16-5.04) | 17 (7.3%) | 38 (3.4%) | 2.55 (1.21-5.35) |
| **Very preterm birth (<32 weeks)** | 7 (2.9%) | 9 (0.8%) | 1.74 (0.22-13.65) | 7 (3.0%) | 9 (0.8%) | 2.05 (0.20-20.95) |
| **Pre-eclampsia** | 15 (6.2%) | 31 (2.7%) | 1.48 (0.65-3.37) | 14 (6.0%) | 30 (2.7%) | 1.45 (0.63-3.32) |
| **Birth weight parameters** |  |  |  |  |  |  |
| **Small for gestational age (SGA)** | 35 (14.6%) | 97 (8.5%) | 1.52 (0.94-2.43) | 34 (14.5%) | 95 (8.4%) | 1.53 (0.95-2.47) |
| **Large for gestational age (LGA)** | 48 (20.1%) | 136 (12.0%) | 1.17 (0.78-1.74) | 47 (20.1%) | 134 (11.9%) | 1.19 (0.80-1.79) |
| **Low birth weight (<2,500 g)** |  |  |  |  |  |  |
| **All live births** | 26 (10.9%) | 38 (3.3%) | 2.66 (1.35-5.24) | 25 (10.7%) | 37 (3.3%) | 3.18 (1.53-6.60) |
| **Term births** | 5 (2.5%) | 16 (1.5%) | 1.25 (0.28-5.48) | 5 (2.6%) | 16 (1.5%) | 1.18 (0.27-5.21) |
| **Missing information** | 1 (0.4%) | 4 (0.4%) |  | 1 (0.4%) | 4 (0.4%) |  |
| **Macrosomia (>4,000 g)** |  |  |  |  |  |  |
| **All live births** | 46 (19.2%) | 233 (20.5%) | 0.72 (0.50-1.04) | 45 (19.2%) | 231 (20.5%) | 0.74 (0.52-1.07) |
| **Term births** | 44 (22.1%) | 233 (21.5%) | 0.79 (0.55-1.15) | 43 (22.1%) | 231 (21.5%) | 0.83 (0.57-1.21) |
| **Other neonatal outcomes** |  |  |  |  |  |  |
| **Apgar <7 at 5 minutes** | 4 (1.7%) | 15 (1.3%) | 1.74 (0.39-7.82) | 4 (1.7%) | 15 (1.3%) | 1.74 (0.39-7.82) |
| **Missing information** | 2 (0.8%) | 9 (0.8%) |  | 2 (0.8%) | 9 (0.8%) |  |
| **Congenital malformations** | 16 (6.7%) | 61 (5.4%) | 1.06 (0.57-2.00) | 14 (6.0%) | 60 (5.3%) | 1.00 (0.52-1.93) |
| **Missing information** | 1 (0.4%) | 4 (0.4%) |  | 1 (0.4%) | 4 (0.4%) |  |
| **Neonatal death** | 1 (0.4%) | 2 (0.2%) | NE | 1 (0.4%) | 2 (0.2%) | NE |
| **All births, n** | 240 | 1140 |  | 235 | 1131 |  |
| **Intrauterine fetal death** |  |  |  |  |  |  |
| **Stillbirth** | 1 (0.4%) | 4 (0.4%) | NE | 1 (0.4%) | 4 (0.4%) | NE |
| **Pregnancy and maternal outcomes** |  |  |  |  |  |  |
| **Induction of labor** | 50 (20.8%) | 159 (13.9%) | 1.27 (0.87-1.85) | 47 (20.0%) | 156 (13.8%) | 1.26 (0.86-1.84) |
| **Cesarean section** | 77 (32.1%) | 182 (16.0%) | 1.63 (1.17-2.27) | 74 (31.5%) | 178 (15.7%) | 1.71 (1.22-2.40) |
| **Elective** | 27 (11.2%) | 83 (7.3%) | 1.18 (0.70-2.00) | 25 (10.6%) | 82 (7.3%) | 1.22 (0.71-2.10) |
| **Emergency** | 45 (18.8%) | 95 (8.3%) | 1.90 (1.20-3.02) | 44 (18.7%) | 92 (8.1%) | 2.06 (1.28-3.31) |
| **Instrumental delivery** | 15 (6.2%) | 83 (7.3%) | 0.84 (0.45-1.59) | 15 (6.4%) | 83 (7.3%) | 0.84 (0.44-1.58) |

Abbreviations: MASLD, metabolic dysfunction-associated steatotic liver disease; n, number; OR, odds ratio; CI, confidence interval; NE, not estimable.

*Model 1: Conditioned on the matching set (maternal age at delivery, calendar year of delivery, and parity).

**Model 2: Conditioned on the matching set and additionally adjusted for level of education, country of birth, BMI in early pregnancy, smoking in early pregnancy, and the following conditions prior to delivery: diabetes (type 1, type 2, and gestational diabetes), hypertension (including gestational hypertension), dyslipidemia, and pre-eclampsia. In sensitivity analysis (restricted to births of women without gestational hypertension), only gestational hypertension prior to pregnancy of interested was included as covariate.

**Table S18:** Pregnancy and birth outcomes for births in women with MASLD versus births in reference women without known MASLD in the main analysis and after restricting to women without pre-eclampsia during the pregnancy of interest (*post-hoc* sensitivity analysis).

|  | **Main analysis** | | | **Sensitivity analysis:**  **Births of women without pre-eclampsia** | | |
| --- | --- | --- | --- | --- | --- | --- |
|  | **Births in women with MASLD**  n (%) | **Births in reference women**  n (%) | **OR (95% CI)**  Model 2** | **Births in women with MASLD**  n (%) | **Births in reference women**  n (%) | **OR (95% CI)**  Model 2** |
| **Live births, n** | 239 | 1136 |  | 224 | 1105 |  |
| **Preterm birth** |  |  |  |  |  |  |
| **Any preterm birth (<37 weeks)** | 40 (16.7%) | 53 (4.7%) | 3.41 (1.98-5.88) | 32 (14.3%) | 46 (4.2%) | 3.41 (1.82-6.37) |
| **Medically indicated** | 23 (9.6%) | 14 (1.2%) | 11.90 (2.46-57.59) | 16 (7.1%) | 9 (0.8%) | 12.93 (2.54-65.87) |
| **Spontaneous** | 17 (7.1%) | 38 (3.3%) | 2.42 (1.16-5.04) | 16 (7.1%) | 36 (3.3%) | 2.61 (1.19-5.74) |
| **Very preterm birth (<32 weeks)** | 7 (2.9%) | 9 (0.8%) | 1.74 (0.22-13.65) | 4 (1.8%) | 8 (0.7%) | 0.00 (0.00-Inf) |
| **Birth weight parameters** |  |  |  |  |  |  |
| **Small for gestational age (SGA)** | 35 (14.6%) | 97 (8.5%) | 1.52 (0.94-2.43) | 29 (12.9%) | 94 (8.5%) | 1.34 (0.81-2.23) |
| **Large for gestational age (LGA)** | 48 (20.1%) | 136 (12.0%) | 1.17 (0.78-1.74) | 45 (20.1%) | 130 (11.8%) | 1.18 (0.78-1.81) |
| **Low birth weight (<2,500 g)** |  |  |  |  |  |  |
| **All live births** | 26 (10.9%) | 38 (3.3%) | 2.66 (1.35-5.24) | 21 (9.4%) | 34 (3.1%) | 3.01 (1.44-6.27) |
| **Term births** | 5 (2.5%) | 16 (1.5%) | 1.25 (0.28-5.48) | 5 (2.6%) | 16 (1.5%) | 1.58 (0.33-7.51) |
| **Missing information** | 1 (0.4%) | 4 (0.4%) |  | 1 (0.4%) | 4 (0.4%) |  |
| **Macrosomia (>4,000 g)** |  |  |  |  |  |  |
| **All live births** | 46 (19.2%) | 233 (20.5%) | 0.72 (0.50-1.04) | 44 (19.6%) | 225 (20.4%) | 0.78 (0.54-1.14) |
| **Term births** | 44 (22.1%) | 233 (21.5%) | 0.79 (0.55-1.15) | 42 (21.9%) | 225 (21.2%) | 0.85 (0.57-1.24) |
| **Other neonatal outcomes** |  |  |  |  |  |  |
| **Apgar <7 at 5 minutes** | 4 (1.7%) | 15 (1.3%) | 1.74 (0.39-7.82) | 4 (1.8%) | 15 (1.4%) | 1.25 (0.25-6.29) |
| **Missing information** | 2 (0.8%) | 9 (0.8%) |  | 2 (0.8%) | 8 (0.7%) |  |
| **Congenital malformations** | 16 (6.7%) | 61 (5.4%) | 1.06 (0.57-2.00) | 13 (5.8%) | 57 (5.2%) | 1.11 (0.56-2.22) |
| **Missing information** | 1 (0.4%) | 4 (0.4%) |  | 1 (0.4%) | 4 (0.4%) |  |
| **Neonatal death** | 1 (0.4%) | 2 (0.2%) | NE | 1 (0.4%) | 2 (0.2%) | NE |
| **All births, n** | 240 | 1140 |  | 225 | 1109 |  |
| **Intrauterine fetal death** |  |  |  |  |  |  |
| **Stillbirth** | 1 (0.4%) | 4 (0.4%) | NE | 1 (0.4%) | 4 (0.4%) | NE |
| **Pregnancy and maternal outcomes** |  |  |  |  |  |  |
| **Induction of labor** | 50 (20.8%) | 159 (13.9%) | 1.27 (0.87-1.85) | 46 (20.4%) | 145 (13.1%) | 1.28 (0.85-1.92) |
| **Cesarean section** | 77 (32.1%) | 182 (16.0%) | 1.63 (1.17-2.27) | 68 (30.2%) | 169 (15.2%) | 1.80 (1.28-2.55) |
| **Elective** | 27 (11.2%) | 83 (7.3%) | 1.18 (0.70-2.00) | 24 (10.7%) | 79 (7.1%) | 1.18 (0.68-2.05) |
| **Emergency** | 45 (18.8%) | 95 (8.3%) | 1.90 (1.20-3.02) | 39 (17.3%) | 87 (7.8%) | 2.35 (1.45-3.83) |
| **Instrumental delivery** | 15 (6.2%) | 83 (7.3%) | 0.84 (0.45-1.59) | 14 (6.2%) | 80 (7.2%) | 0.82 (0.42-1.60) |

Abbreviations: MASLD, metabolic dysfunction-associated steatotic liver disease; n, number; OR, odds ratio; CI, confidence interval; NE, not estimable.

*Model 1: Conditioned on the matching set (maternal age at delivery, calendar year of delivery, and parity).

**Model 2: Conditioned on the matching set and additionally adjusted for level of education, country of birth, BMI in early pregnancy, smoking in early pregnancy, and the following conditions prior to delivery: diabetes (type 1, type 2, and gestational diabetes), hypertension (including gestational hypertension), dyslipidemia, and pre-eclampsia. In sensitivity analysis (restricted to births of women without pre-eclampsia), only pre-eclampsia prior to pregnancy of interested was included as covariate.
